# Dry Priming of Maize Seeds Reduces Aluminum Stress

**DOI:** 10.1371/journal.pone.0145742

**Published:** 2015-12-29

**Authors:** Berenice Kussumoto Alcântara, Katja Machemer-Noonan, Francides Gomes Silva Júnior, Ricardo Antunes Azevedo

**Affiliations:** 1 Departamento de Genética, Escola Superior de Agricultura “Luiz de Queiroz”, Universidade de São Paulo, Piracicaba, São Paulo, Brasil; 2 Center for Applied Plant Sciences, Rightmire Hall, The Ohio State University, Columbus, Ohio, United States of America; 3 Departamento de Ciências Florestais, Escola Superior de Agricultura “Luiz de Queiroz”, Universidade de São Paulo, Piracicaba, São Paulo, Brasil; Wuhan Botanical Garden, Chinese Academy of Sciences, CHINA

## Abstract

Aluminum (Al) toxicity is directly related to acidic soils and substantially limits maize yield. Earlier studies using hormones and other substances to treat the seeds of various crops have been carried out with the aim of inducing tolerance to abiotic stress, especially chilling, drought and salinity. However, more studies regarding the effects of seed treatments on the induction of Al tolerance are necessary. In this study, two independent experiments were performed to determine the effect of ascorbic acid (AsA) seed treatment on the tolerance response of maize to acidic soil and Al stress. In the first experiment (greenhouse), the AsA seed treatment was tested in B73 (Al-sensitive genotype). This study demonstrates the potential of AsA for use as a pre-sowing seed treatment (seed priming) because this metabolite increased root and shoot growth under acidic and Al stress conditions. In the second test, the evidence from field experiments using an Al-sensitive genotype (Mo17) and an Al-tolerant genotype (DA) suggested that prior AsA seed treatment increased the growth of both genotypes. Enhanced productivity was observed for DA under Al stress after priming the seeds. Furthermore, the AsA treatment decreased the activity of oxidative stress-related enzymes in the DA genotype. In this study, remarkable effects using AsA seed treatment in maize were observed, demonstrating the potential future use of AsA in seed priming.

## Introduction

Acidic soil (pH < 5.5) constitutes an abiotic stress that significantly affects the yield of agricultural crops worldwide [1]. A striking characteristic of acidic soil is the presence of aluminum ions (Al^3+^), which trigger a series of symptoms known as aluminum (Al) toxicity syndrome in plants [2]. Al toxicity in acidic soils occurs as a result of excess hydrogen disrupting the crystal structure of soil particles, followed by the release of Al^3+^ ions, allowing them to attach to sites of cation exchange and substitute cationic nutrients, such as calcium [3]. Additionally, it has been shown that Al may interfere with core-related processes in the cell, such as DNA synthesis [4] and cell division [5]. In maize, Al causes oxidative stress [6], reduces photosynthetic capacity and electron transport rates, and negatively changes the accumulation of nutrients in the shoots [7]. In maize, this problem can cause yield losses of up to 80% [8].

The experiments described in this study were performed in two of the world’s leading producers of maize, the United States (313.9 million tons per year (mt/y)) and Brazil (55.6 mt/y), both possessing large agricultural areas in regions with exchangeable Al associated with acidic soils [9,10]. However, with regard to the average productivity, these countries are not ranked in the top positions [10]. One factor that could restrict agricultural productivity in the United States and Brazil is the presence of Al^3+^ in acidic soils. This is because approximately 50% of the potential arable lands in the U.S. have soil with Al^3+^ toxicity, and 20% of all maize that is planted in this country is found in regions with this type of soil [9]. In Brazil, the data are even more extreme because approximately 97% of the potential arable land exhibits the problem of exchangeable Al in the soil [10], leading to lime application to enable agricultural production in such acidic soils [11–13]. However, without abundant organic matter, the effect of lime is observed only in the first 10 cm of the soil and extends to approximately 20 cm when bound to organic waste [14]. Nonetheless, it is important to consider that lime is also a non-renewable natural resource that can eventually be exhausted.

Previous studies show that some plant species are tolerant of phytotoxic amounts of Al [15,16] and differences exist even among cultivars of the same species [17]. In maize, for example, the SA-3, SA-8 [18], C100-6 [6] and Cateto-Colombia [19] cultivars belong to the genetically tolerant varieties, whereas S1587-17 [6], B73, L53 [20], Tuxpeño [21] and Mo17 [19] represent Al-sensitive maize varieties.

According to Kochian et al. [1] and references therein, two mechanisms of Al tolerance can be encountered in plants: internal tolerance and exclusion. When the ions are tolerated in the cytosol, the mechanism is defined as an internal mechanism, which could involve complexation of Al^3+^ with polypeptides [22] and compartmentalization of Al in the vacuole [2]. Exclusion mechanisms involve immobilization of Al ions in cell walls by complex formation with lignin [23], whereas relatively high rhizosphere pH levels cause Al to remain insoluble [24]. Further, the release of organic acids, such as malate and citrate [25], which complex with Al in the rhizosphere, is considered to be the most effective strategy [26]. However, Kochian et al. [1] and Piñeros et al. [20] showed that the Al-sensitive maize variety Mo17 produced the highest levels of citrate, indicating that other mechanisms of Al tolerance could act simultaneously in plants.

The production of lignin in roots may also be related to Al tolerance. Ezaki et al. [23] observed that increased production of lignin in transgenic *Arabidopsis thaliana* roots enhanced Al tolerance. Kumari et al. [27] analyzed the transcriptional response of *A*. *thaliana* plants under Al stress, detecting 152 cell wall-related genes, among which caffeic acid O-methyltransferase (*COMT*), the key gene of lignin biosynthesis [28], presented increased abundance of transcripts.

In maize, two transcription factors belonging to the R2R3-type MYB gene family may be involved in the repression of genes related to lignin biosynthesis, i.e., ZmMYB31 and ZmMYB42 [29,30]. Over-expression of *ZmMYB*31 in *A*. *thaliana* plants caused repression of the genes related to lignin synthesis, including *COMT* [29]. Generally, members of the MYB transcription factor family are responsible for regulating secondary metabolism, cell growth and shape, and the responses to hormones and some stresses, such as drought and viral infection [31–33]. Therefore, characterization of the expression of *COMT* and its transcriptional regulators under conditions of oxidative stress represents a relevant research topic that aims to provide a better understanding of how lignin biosynthesis may be affected.

In addition to genetic tolerance, further studies have established a means to diminish the effect of abiotic stresses using conditioning of seeds prior to germination under stress conditions [34–37], e.g., cold tolerance induced by seed priming with KCl [36], acquired salinity tolerance using seed priming with chloride salts or ethylene [34,35], and drought resistance induced by ascorbic acid (AsA) [37]. However, these cited treatments were performed under wet conditions. Dry seed treatments could be agriculturally relevant as observed for insecticides [38] and fungicides [39] that are applied to seeds for alleviation of the effects of biotic stresses.

Pre-sowing seed treatment techniques (a.k.a. seed priming) increase the synchronization of germination and improve plant vigor by activating key enzymes, including amylases, proteases and lipases essential for early growth and development of embryos [40], and by increasing gene activation related to the minimization of stress conditions [41,42]. Antioxidant defenses evolved in plants to minimize the harmful effects of reactive oxygen species (ROS) generated from oxygen, including non-enzymatic compounds, such as ascorbic acid and also enzymatic systems, such as those including catalase (CAT), ascorbate peroxidase (APX) and guaiacol-type peroxidases (GPOX), among others enzymes [43,44]. In an optimal environment, the oxidant/antioxidant system is balanced. However, under conditions where an excess of ROS is present due to stimuli that may include, for example, ozone [45], heavy metals [46,47], mineral deficiencies [7], water-deficit stress [48,49] and Al [6], an oxidative stress condition may occur. An imbalance in the redox state of the cell is deleterious to cell integrity and metabolism and must be ameliorated to enable the organism to tolerate the stimuli and grow.

Currently, more studies are necessary to verify the effect of seed priming on tolerance induction to Al stress in maize. Previous evidence suggested that root treatment with exogenous AsA can ameliorate root growth when 2-day-old rice seedlings are exposed to AlCl_3_ [50], showing the potential of this treatment to minimize Al stress. Furthermore, the increase of AsA biosynthesis enhanced stress resistance in yeast [51]. This study aimed to test AsA powder as a dry seed-priming technique for maize under Al stress. This work is a novel approach that differs from typical seed priming under wet conditions. The objective of this study was to determine how this treatment affects lignin biosynthesis and stress-related enzymes, and consequently, its effect on maize productivity.

## Materials and Methods

### Plant material

Seeds of the maize (*Zea mays* L.) inbred line B73 (Al-sensitive) were used in a greenhouse experiment that was performed under laboratory conditions in the United States (The Ohio State University, Columbus, OH). The Al-sensitive maize line (Mo17) and the commercial simple hybrid 2B587PW (Al-tolerant from Dow AgroSciences, abbreviated as DA in this study) were used in the experiment conducted under field conditions in Brazil (Universidade de São Paulo, Escola Superior de Agricultura “Luiz de Queiroz”, Estação Experimental do Departamento de Genética, Anhumas district, SP) because it was not possible to obtain B73 seeds for this experiment.

### Dry seed priming

Seeds for the greenhouse and field experiments were dry-primed as follows: AsA-primed seeds were coated with AsA at a ratio of 3.8 g AsA powder per 25 seeds (sufficient amount to cover all seeds). The seeds were placed in Falcon tubes and shaken with AsA in order to cover all the seeds with powder. After treatment, the seeds were sieved to remove excess powder and planted and cultivated under greenhouse or field conditions. In order to compare our study with others, 100 mM of AsA was estimated to be entering in the seeds since around 1.8 mg per seed was adhered to surface of B73 seeds. Seeds were imbibed until constant weight (maximum imbibition) obtaining 0.1 g of water per seed; the measurements were carried out in intervals of 1 hour until constant weight. Assuming 100% of adhered AsA entered the seed, this is approximately equivalent to imbibing a seed in 100 mM.

### Greenhouse experiment

In this experiment, L-ascorbic acid (AsA) was tested under greenhouse conditions. Five repetitions and two seeds per vase were used. Each vase was considered one repetition. The seeds were germinated under a photoperiod of 16 hours of light at a temperature of 25°C. For the control, unprimed seeds of the inbred line B73 were germinated in soil at pH 6.0 with 2 mmol_c_.dm^3^ Al (considered not harmful) or in soil at pH 4.0 with 6 mmol_c_.dm^3^ Al (considered slightly harmful).

Dry AsA-primed seeds of the inbred line B73 were planted in soil at pH 6.0 with 2 mmol_c_.dm^-3^ Al (not harmful) or in soil at pH 4.0 with 6 mmol_c_.dm^-3^ Al (slightly harmful) for germination.

#### Evaluation of growth and determination of Al content

The lengths of the roots and shoots were evaluated 5 days after germination and measured with a ruler. For the roots, the longest measurements were considered. In order to perform gene expression analysis, damage to the roots was avoided by preventing drastic root uncoiling. Only the root tips were stretched and the root was carefully measured from the beginning until the tip. Two plants per repetition were used, one plant of each repetition was used for RNA extraction and the other plant was used for Al quantification. The absorption of Al in both tissue types was determined via inductively coupled plasma optical emission (ICP-OES, Optima 3000 DV, Perkin Elmer, Trace Element Research Laboratory, OH, USA). The roots and shoots were dried at 60°C, ground to fine powder, and 0.1 g of the vegetal material was digested in pure nitric acid. After acid digestion, the extract was diluted to 10% (v/v) in deionized water and was immediately used for ICP-OES determination.

#### RNA extraction and cDNA synthesis

Fresh material (roots and shoots) was frozen and ground under liquid nitrogen. Approximately 100 mg of ground frozen material was used for total RNA extraction (#74904, RNeasy Plant Mini Kit, Qiagen). After RNA extraction, total RNA was treated with DNase (On-Column DNase I Digestion Set, DNASE70-1SET, Sigma) for 20 min at room temperature. Total RNA was quantified using a NanoDrop ND-1000 UV/VIS spectrophotometer (NanoDrop Technologies). Approximately 100 ng of total RNA was used to generate cDNA using an Invitrogen kit (SuperScript II). Oligo-dT primers were used to bind the polyA tails of the mRNAs to initiate the synthesis of first-strand cDNA with reverse transcriptase. The obtained cDNA samples were treated with RNase. Amplification through conventional PCR was conducted in a final volume of 25 μl, containing 2.5 μl of 10x buffer supplied with Mg^2+^ (E00007, GeneScript), 0.2 mM dNTPs, 1.0 μM forward primer, 1.0 μM reverse primer, 0.05 U/μl Taq polymerase and 100 ng of cDNA. The reactions were performed in a Peltier Thermal Cycler, model PTC-225 (MJ Research, Inc.) using the following amplification conditions: denaturation at 94°C for 2 min followed by 35 cycles at 94°C for 30 s, a melting temperature of 57°C for 45 s and 70°C for 1 min, with a final extension at 70°C for 10 min.

#### qRT-PCR expression analysis

Quantitative real-time PCR (qRT-PCR) was performed at the Plant-Microbe Genomics Facility (The Ohio State University, Columbus, OH, United States) using the following protocol for Bio-Rad CFX96: step 1: 95.0°C for 10 min; step 2: 95.0°C for 15 s; step 3: 60.0°C for 1 min; step 4: steps 2–3 49 more times; step 5: melt curve of 45.0°C to 95.0°C, with an increase of 0.5°C every 5 s. The following primers were used for qRT-PCR expression analysis: UBQ accession number GRMZM2G118637 (www.maizesequence.org) (F: GTGAGTCGTGACTGAGCTGGTT; R: ATATGCGGTCGCACGATAGTT), COMT1 accession number M73235 (F: TCTGCGTCGAATTGTCTCTGC; R: GAGAGCAATTAAACCGCCATGT), ZmMYB31 accession number GRMZM2G050305 (F: GTAGGTGAAAAATACGCGATGG; R: AAAATGGAGCAGCAAGAGAGAG) and ZmMYB42 accession number GRMZM2G419239 (F: CTGGGGCTCAGGACCAGCGT; R: GGGAGCAGCTACTGTGGGGAGG).

The analyses were performed using the roots and shoots of five-day-old maize seedlings. For each treatment, the relative gene expression (*RGEx*) was calculated using the formula adapted from Morohashi and Grotewold [52], where Ct*rg* is the Ct value for the reference gene (*UBQ*) and Ct*pg* is the Ct value for the phenylpropanoid-related gene. Three biological repetitions and three technical replicates were used for the analysis of gene expression.

### Field experiment

The field experiment was performed in a soil of a cerrado region (Brazilian savanna—naturally high in Al content) in the Anhumas district (Lat. 22°45'–22°50' S; Long. 48°00'–48°05' W; 460 m altitude). This location belongs to an experimental area of the University and no specific permissions were required for conduct the experiment. The field experiment did not involve any contaminations of the environment.

For the field experiment, seeds of the maize (*Zea mays* L.) inbred line Mo17 (Al-sensitive) and the commercial simple hybrid DA (Al-tolerant) were used. Dry AsA-primed seeds of both genotypes were planted using a randomized block design with five repetitions, and covering a total area of 690 m^2^. Each block was composed of 3 rows (8 plants per row), and to guarantee the germination of all plants per row with the aim of analyzing the productivity, 2 seeds were sown per fissure. The seeds were sown in summer 2013.

The thinning step occurred after 50 days of germination. The kernels were harvested in winter (dry season) 2013. Unprimed and AsA-primed seeds were cultivated in a Yellow Red Latosol (Oxisol) soil with the following characteristics: pH 4.6; 729 mmol_c_.dm^-3^ Al (considered very harmful); 18 mg Kg^-1^ phosphorus; 2.6 mmol_c_ Kg^-1^ potassium; 5 mmol_c_ Kg^-1^ calcium; 3 mmol_c_ Kg^-1^ magnesium; 19% base saturation; and 72% changeable aluminum. The soil was supplemented with nitrogen, phosphorus and potassium using NPK 08:28:16 250 Kg ha^-1^ prior to sowing. For this experiment, the soil was not limed to maintain the acidic characteristic to test the efficiency of the AsA treatment on the maize seeds.

#### Maize growth and grain yield

When yield parameters are concerned, the following measurements were carried out: total height (cm) for Mo17 and DA at 70 days after sowing (before Mo17 death); weight of 1000 kernels (g) and grain yield (t ha^-1^) harvested at 167 days after sowing only for DA (Mo17 died after 104 days). To obtain the grain yield, all maize ears in each block were harvested and using random sampling, 35 cobs from each treatment were obtained. The weight of 1000 kernels was measured and the mean grain number per block was estimated.

#### Nutrient and Al uptake

Maize shoots were collected during the thinning step (50 days old) from 3 blocks composed of 8 plants. The samples were dried at 60°C and sent to the Laboratório de Análise de Tecido Vegetal at Departamento de Ciências do Solo of “Escola Superior de Agricultura Luiz de Queiroz–Universidade de São Paulo”. The nutrient content was measured according to Malavolta et al. [53].

#### Lipid peroxidation

The rate of oxidative damage of the maize leaves was carried out during thinning (50 days old) and quantified biochemically as described previously [54]. Metabolites that were reactive to 2-thiobarbituric acid (TBA) were used to estimate the levels of malondialdehyde (MDA), which is an indicator of lipid peroxidation [55]. Readings at 535 and 600 nm were measured using a spectrophotometer, and the concentration of MDA was determined using the following equation: *C* = [*ABS*(535–600)÷155,000]x10^3^. The results were expressed as μmol MDA∙g^-1^ fresh matter. Three independent replicates, each composed of the leaves of 8 plants, were used.

#### Hydrogen peroxide

Hydrogen peroxide levels were measured in maize leaves collected during thinning (50 days old) using the method described by Alexieva et al. [56]. Maize leaves were homogenized in 0.1% (w/v) trichloroacetic acid (TCA). The homogenate was centrifuged at 12,000 × *g* for 15 min at 4°C. Following centrifugation, 200 μL of supernatant was added to 200 μL of 100 mM potassium phosphate buffer (pH 7.0) and 800 μL of 1 M KI. The readings were taken at 390 nm. Three independent replicates, each composed of the leaves of 8 plants, were used.

#### Ascorbate quantification

The ascorbate content was determined as described by Arakawa et al. [57] with modifications. Maize leaves were homogenized in 5% (w/v) TCA. The homogenate was centrifuged at 15,000 × *g* for 15 min at 4°C. Following centrifugation, 50 μL of supernatant was collected and diluted in 70 μL of TCA (5%), 125 μL of pure ethanol and 125 μL of Na_2_HPO_4_ (0.2 M, pH 8.0). This mixture was incubated at 25°C for 10 min. Subsequently, the following reagents (all dissolved in pure ethanol) were added to the same tube: 125 μL of 0.24% (w/v) N-ethylmaleimide, 125 μL of pure ethanol, 125 μL of 4% (v/v) H_3_PO_4_, 250 μL of 0.5% (w/v) bathophenanthroline and 150 μL of 0.03% (w/v) FeCl_3_. The tubes were vortexed, and the mixture was incubated at 30°C for 90 min. The readings were taken at 534 nm. Three independent replicates, each composed of the leaves of 8 plants were used.

#### Antioxidant enzyme extraction

Maize leaves were collected from just below the flag leaf of 50-day-old plants, macerated in liquid nitrogen and homogenized using a mortar and pestle in 100 mM potassium phosphate buffer (pH 7.5) containing 1 mM EDTA, 3 mM DTT and 4% (w/v) insoluble PVPP [43] at a ratio of 5:1 (buffer volume:fresh weight). The homogenate was centrifuged at 10,000 × *g* for 30 min at 4°C [44], and the supernatant was stored in 200 μL aliquots at -80°C for catalase (CAT, EC 1.11.1.6), guaiacol-type peroxidase (GPOX, EC 1.11.1.7), ascorbate peroxidase (APX, EC 1.11.1.11) and glutathione reductase (GR, EC 1.6.4.2) activity determination. The protein concentrations of all samples were determined using the Bradford [58] method.

#### Polyacrylamide gel electrophoresis (PAGE)

Electrophoretic analysis was carried out under non-denaturing conditions in 10% polyacrylamide gels, followed by specific enzyme activity staining for CAT, GR and APX as described in the next sections, with 20 μg of protein being loaded onto each gel lane for all situations and treatments. The electrophoresis buffers and gels were prepared as described by Medici et al. [59].

#### Enzyme assays

**Catalase (CAT):** CAT activity staining in native PAGE gels was determined as described by Medici et al. (2004). The gels were incubated in 0.003% H_2_O_2_ for 10 min and developed in a 1% (w/v) FeCl_3_ and 1% K_3_Fe(CN_6_) (w/v) solution for 10 min. Densitometry was performed using ImageJ software, version 1.47 (National Institutes of Health, U.S.A; http://imagej.nih.gov/ij). The relative percentage was calculated based on the activity of the standard in the gel (CAT from bovine liver, Sigma C-100 lot 20K7010).

**Glutathione reductase (GR):** GR activity staining in native PAGE gels was determined as described by Medici et al. (2004). The gels were incubated in the dark for 30 min at room temperature in 50 mL of a reaction mixture containing 250 mM Tris buffer (pH 7.5), 3.4 mM GSSG, 0.5 mM NADPH, 50 mg of 3-(4,5-dimethyl-2-thiazolyl)-2,5-diphenyl-2H-tetrazolium bromide (MTT) and 10 mg of 2,6-dichloro-N-(4-hydroxyphenyl)-1,4-benzo-quinoneimine sodium salt (DPIP). The relative percentage was calculated based on the activity of the standard in the gel (GR from yeast, Sigma G3664).

**Ascorbate peroxidase (APX):** APX activity staining in native PAGE gels was determined according to Mittler and Zilinskas [60] and Erdal [61]. The gels were pre-run for 30 min with carrier buffer plus 2 mM ascorbate. Following electrophoretic separation, the gels were incubated with 50 mM sodium phosphate buffer (pH 7.0) containing 4 mM ascorbate and 2 mM H_2_O_2_ for 20 min. APX activity was observed after the gels were submerged in a solution of 50 mM sodium phosphate buffer (pH 7.8), 28 mM TEMED and 2.45 mM NBT. The relative percentage was calculated from the identified bands.

**Guaiacol-type peroxidase (GPOX):** GPOX total activity was evaluated by spectrophotometry as described by Gratão et al. [46] with some modifications. The reaction mixture, which contained 390 μL of phosphate-citrate buffer, pH 5.0 (0.2 M dibasic sodium phosphate:0.1 M citric acid), 10 μL of enzyme extract and 25 μL of 0.5% guaiacol, was vortexed and incubated in a water bath at 30°C for 15 min. The reaction was terminated by cooling in an ice-water bath, followed by the addition of 25 μL of 2% sodium metabisulfide. The reaction mixture was held for 10 min and the GPOX activity was evaluated by monitoring the absorbance at 450 nm.

#### Klason lignin determination

For Klason lignin determination, dried maize shoots that were obtained during thinning (50 days old) were ground using a mill knife and were passed through a 60-mesh sieve. The results were expressed relative to 1 g of completely dried material. Plants from the thinning step were used for lignin analysis; therefore, the shoots were cut and the neighbor plants were left in the field for productivity analysis. The whole plant (with roots and shoots) was not removed to avoid damaging the roots of nearby plants. For this reason, the lignin in roots was not analyzed to maintain the roots of neighboring plants in a good condition for maize production.

For acid hydrolysis, 300 mg of dried sample without extract was used to which 3 mL of 72% H_2_SO_4_ was added. This mixture was incubated in a water bath at 30°C for 1 hour. At the end of this period, 84 mL of deionized water was added and the flasks were placed in an autoclave at 120°C for 1 hour. After acid hydrolysis, the samples were filtered using glass microfiber (GF-1; D 47 mm), and the acid-insoluble lignin was determined gravimetrically; the acid-soluble lignin was determined spectrophotometrically at 215 nm and 280 nm [62].

### Statistical analysis

Statistical analyses for all data were performed using StatSoft software (version 7.0, Tulsa, OK, USA). The significant differences between the means of the treatments were determined at a confidence level of 95% using Duncan’s test.

## Results

### Greenhouse experiment

**Fig 1:**
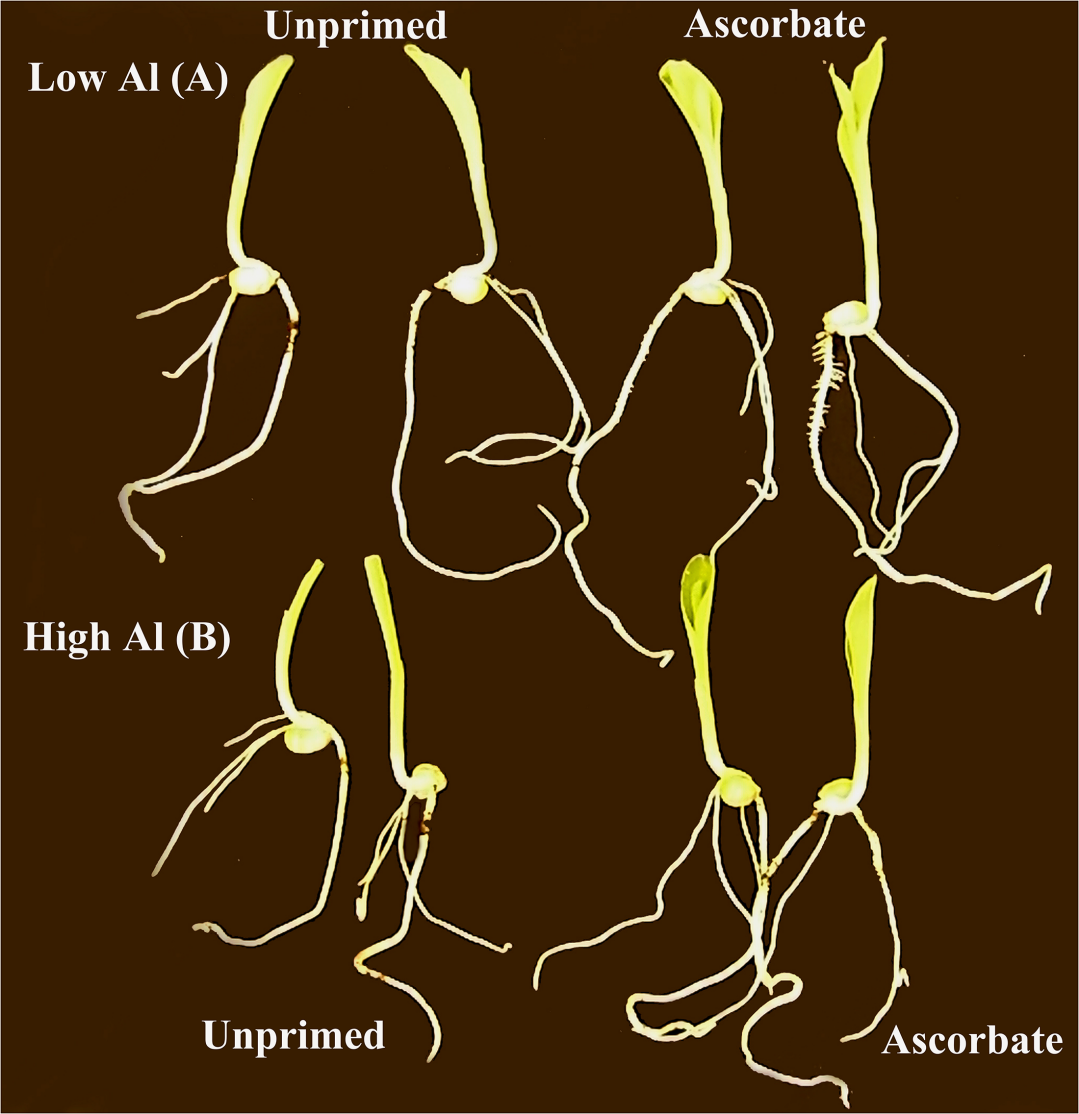
Growth comparison of 5-day-old maize seedlings (B73). Unprimed and AsA-primed maize seeds that germinated in optimum soil (A) and unprimed and AsA-primed maize seeds that germinated in acidic soil with aluminum stress (B).

**Fig 2:**
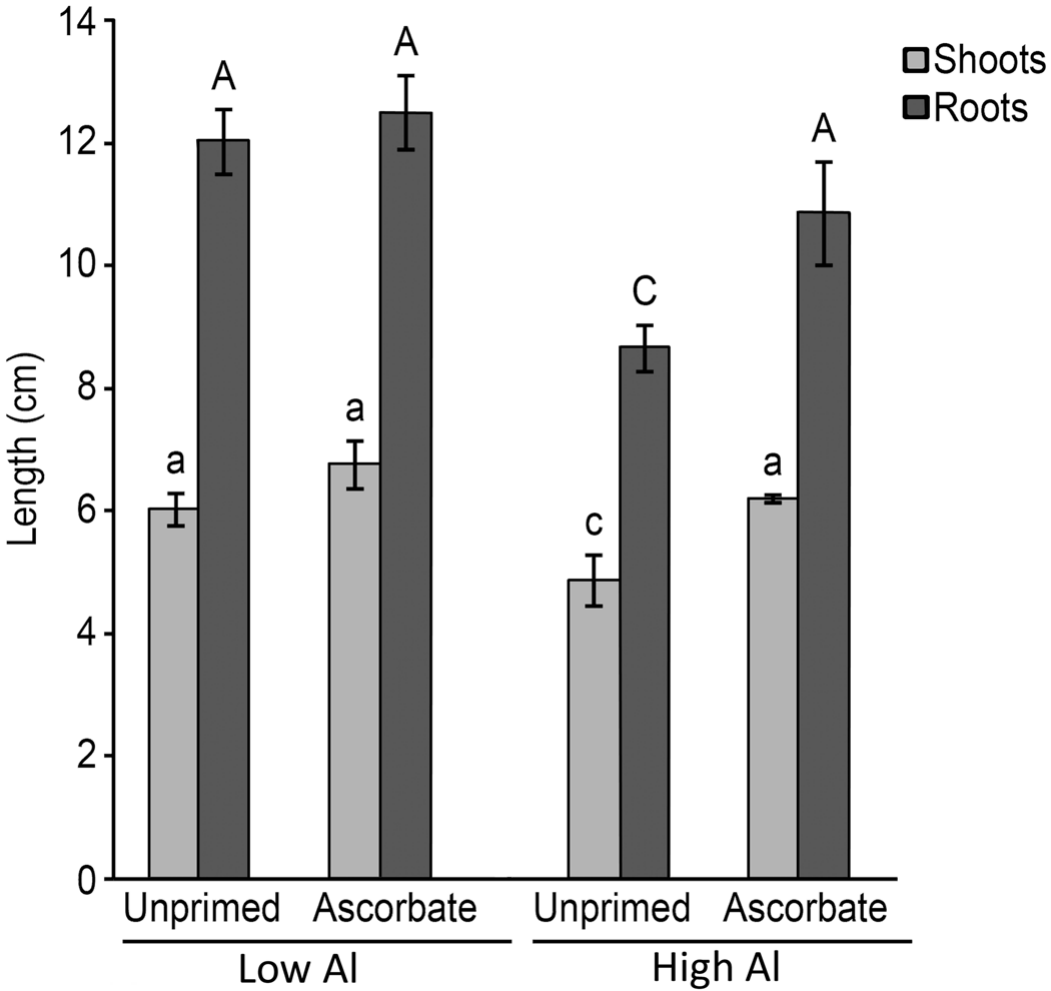
Length of roots and shoots of 5-day-old maize seedlings (B73). Different uppercase letters denote significant differences between roots (dark gray). The significant differences between shoots (light gray) are shown by different lowercase letters (p < 0.05).

Al toxicity in acidic soils is a worldwide concern as this problem causes inhibition of root and shoot growth [1,2]. The AsA treatment significantly (p < 0.05) improved the growth of the roots and shoots when germination occurred under Al stress (Fig 1). The seeds treated with AsA and germinated in soil at pH 6.0 did not exhibit any difference in the lengths of the roots and shoots (12.0 cm and 6.0 cm, respectively) compared with those of the untreated seeds (12.5 cm and 6.8 cm, respectively) (Fig 2). However, in soil at pH 4.0, the AsA-primed seeds exhibited longer roots (10.9 cm) and shoots (6.2 cm) compared with those of the unprimed seeds (8.7 cm and 4.9 cm, respectively) (Fig 2).

**Fig 3:**
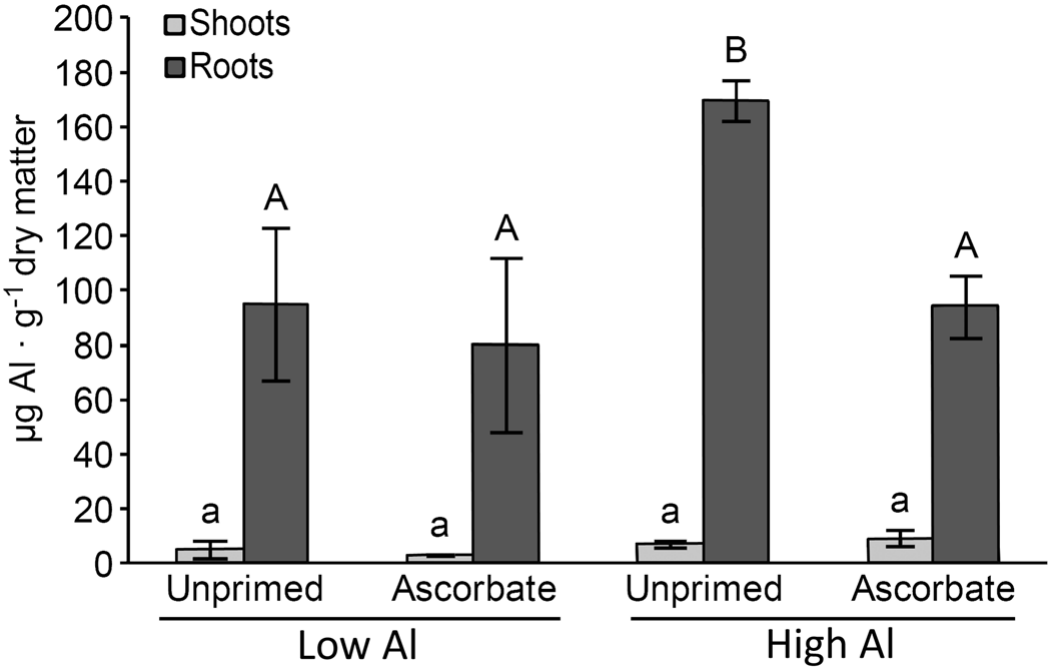
Quantification of aluminum in 5-day-old maize seedlings (B73). Different uppercase letters denote significant differences between roots (dark gray). The significant differences between shoots (light gray) are shown using different lowercase letters (p < 0.05).

The analysis of the Al content showed that unprimed seeds sown under stress exhibited higher Al uptake by roots compared with the seeds primed with AsA (Fig 3). Furthermore, the AsA seed treatment reduced the uptake of Al by the root (from 169.53 to 94.09 μg Al.g^-1^ dry matter) (Fig 3). A small amount of Al was detected in the shoots; however, no treatment differences were observed (Fig 3).

**Fig 4:**
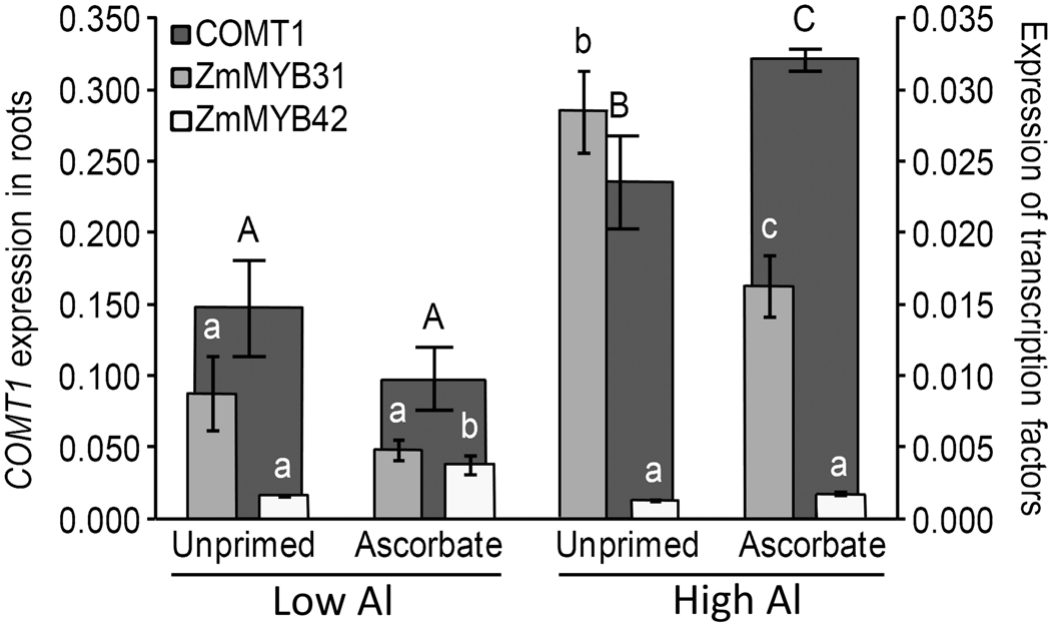
*COMT*1, *ZmMYB*31 and *ZmMYB*42 expressions in the roots of 5-day-old maize seedlings (B73). Different uppercase letters denote significant differences between *COMT*1 gene expressions (dark gray). The expression differences of the transcription factors *ZmMYB*31 (light gray) and *ZmMYB*42 (white) are shown using different lowercase letters (p < 0.05).

**Fig 5:**
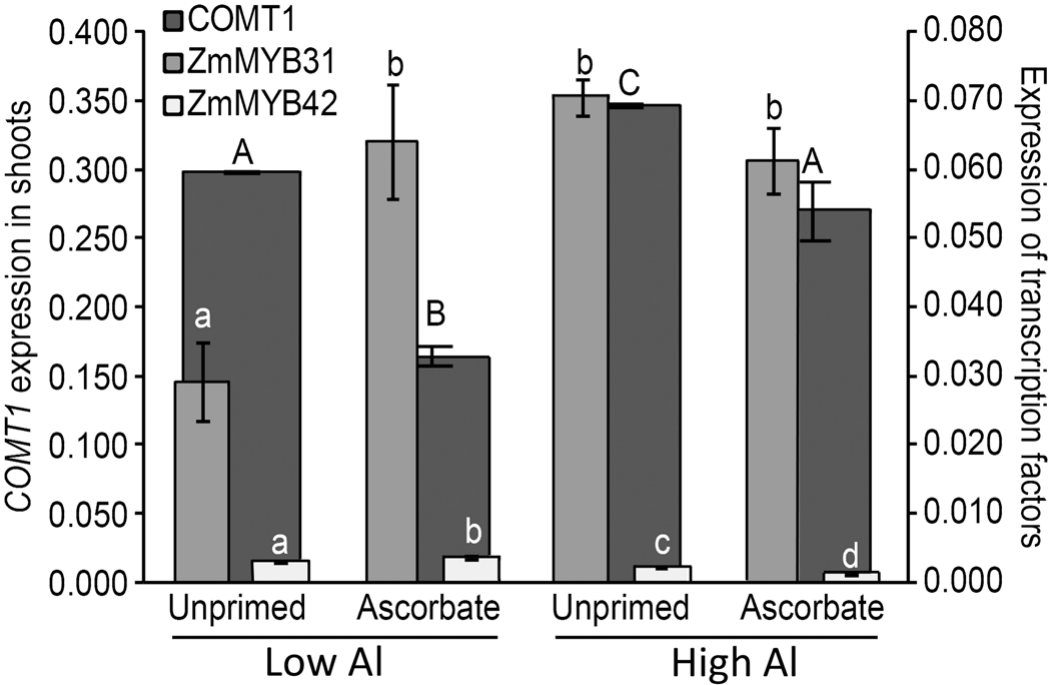
*COMT*1, *ZmMYB*31 and *ZmMYB*42 expressions in the shoots of 5-day-old maize seedlings (B73). Different uppercase letters denote significant differences between *COMT*1 gene expressions (dark gray). The expression differences of the transcription factors *ZmMYB*31 (light gray) and *ZmMYB*42 (white) are shown using different lowercase letters (p < 0.05).

Up-regulation of lignin gene expression in the roots has been linked to Al tolerance in previous work [23,63]. It was observed that in the roots of unprimed seeds, the key lignin gene, *COMT*1, was significantly over-expressed (p < 0.05) when the seeds were germinated in acidic soil (the relative gene expression increased from 0.148 to 0.235) (Fig 4). Notably, the expression of *COMT*1 in the roots was much higher when seeds were treated with AsA and germinated in acidic soil (0.321), however, the effect on *COMT*1 expression in the roots (Fig 4) was opposite to that of the shoots (Fig 5), resulting in decreased expression. Ascorbic acid reduced the expression of *COMT*1 when germination occurred in low Al (from 0.299 to 0.165) and in high Al (from 0.347 to 0.270) (Fig 5). A summary of Al content and gene expression data is found in Table 1.

**Table 1:** Summary of Al content and gene expression data.

Effect of AsA priming* on	Root (low Al)	Root (high Al)	Shoot (low Al)	Shoot (high Al)
Al content	Neutral	Decreased	Neutral	Neutral
*ZmMYB*31	Neutral	Decreased	Increased	Neutral
*ZmMYB*42	Increased	Neutral	Increased	Decreased
*COMT*1	Neutral	Increased	Decreased	Decreased

*compared to control plants.

Two transcription factors are thought to be related to the repression of genes involved in lignin biosynthesis, i.e., ZmMYB31 [29] and ZmMYB42 [30]. The expression of the transcription factor ZmMYB31 was reduced in the roots when the seeds were treated with AsA and germinated under stress (Fig 4). The relative gene expression in the roots under stress was 0.029 for the unprimed seeds and 0.016 for the primed seeds. However, in the shoots, the treatment with AsA did not alter the expression of *ZmMYB*31 under acidic soil and Al stress (Fig 5). Curiously, the expression of another possible repressor, *ZmMYB*42, was significantly reduced in the shoots under acidic soil and Al stress (Fig 5).

### Field experiment

**Fig 6:**
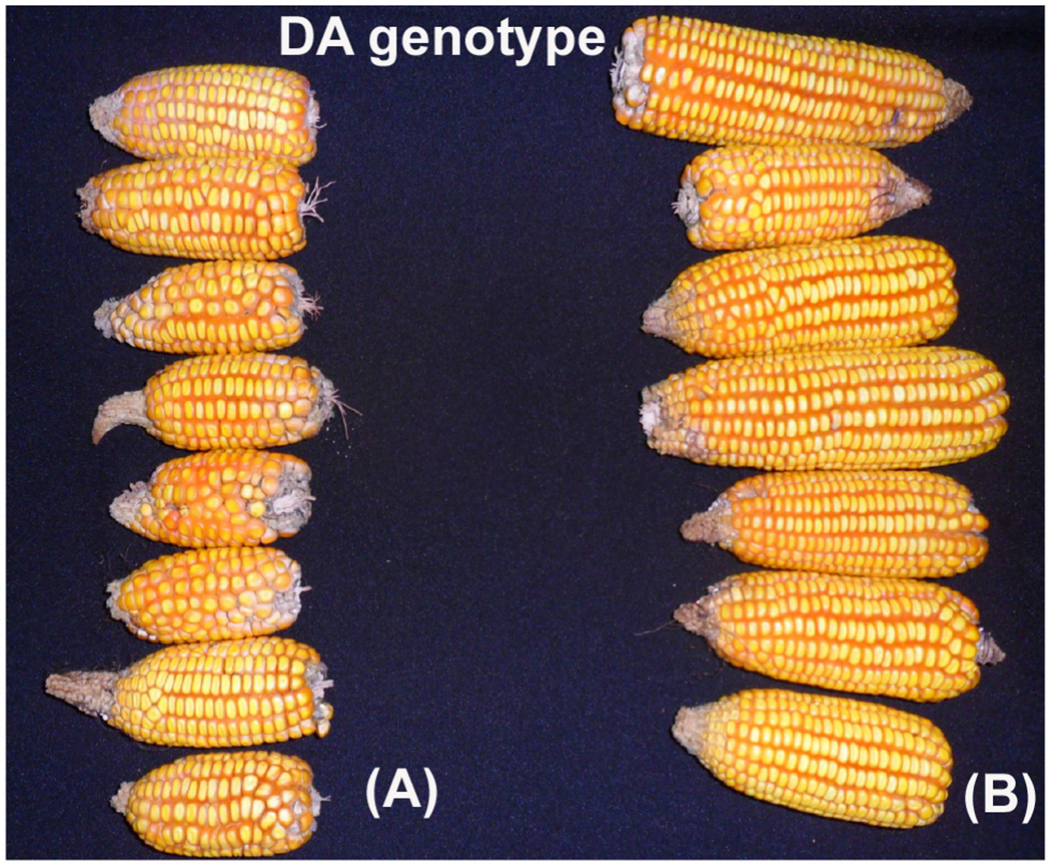
Differences in the maize cob sizes of DA plants. Maize cobs that originated from unprimed seeds (A) and maize cobs that originated from AsA-primed seeds (B).

Grain yield was analyzed for DA plants only, as all Mo17 plants died within 104 days after sowing (Table 2). The total height was measured before Mo17 death (70 days after sowing). The mean total height for DA plants of unprimed seeds was 120.56 ± 9.57 cm, whereas AsA-primed seeds were 150.99 ± 6.19 cm on average. For the sensitive line Mo17, the mean total height of the plants of unprimed seeds was 37.96 ± 7.15 cm compared with 52.02 ± 6.98 cm for AsA-primed seeds. In general, growth inhibition was observed in Mo17, which is the major symptom of Al stress. Ascorbic acid seed treatment only improved the productivity of the DA genotype as shown in Fig 6.

**Table 2:** Productivity parameters for the maize genotypes. Different uppercase letters denote significant differences between the genotypes. The significant differences among seed treatments are shown using different lowercase letters (n = 5).

Genotype	Seed treatment	Total height (cm)*	Weight of 1000 kernels (g)	Number of grains per block	Grain yield (t.ha^-1^)
**DA**	Unprimed	120.57 aA	263.98 a	15701 a	1.63 a
	Ascorbate	150.99 bA	339.02 a	19953 b	2.65 b
	CV (%)	21.11	16.88	6.37	22.2
**Mo17**	Unprimed	37.96 cB	-	-	-
	Ascorbate	52.03 dB	-	-	-
	CV (%)	21.11	-	-	-

* Total height of maize plants at 70 days after sowing.

Variations in the number of grains per block (15701 grains for kernels originating from the unprimed seeds and 19953 grains for kernels originating from the primed seeds) were observed (Table 2). Furthermore, differences in the grain yield per hectare were also observed, i.e., 1.63 t.ha^-1^ for the kernels originating from the unprimed seeds and 2.65 t.ha^-1^ for the kernels originating from the seeds that were primed with AsA (Table 2). However, there was no difference in the weight of 1000 kernels (263.98 g for kernels originating from the unprimed seeds and 339.02 g for kernels originating from the primed seeds) (Table 2).

#### Nutrient and Al uptake

Maize shoots obtained during the thinning step (50 days old) were sampled, and nutrient analysis showed that magnesium and manganese were taken up more by DA than by Mo17 plants (Table 3). However, the amounts of phosphorus and sulfur were higher in Mo17 than in DA plants. When comparing the genotypes within a treatment, the amount of Al was higher in Mo17 than in DA (215.30 versus 99.33 mg Al.Kg^-1^ dry matter for unprimed seeds and 173.80 versus 107.07 mg Al.Kg^-1^ dry matter for AsA-primed seeds). Surprisingly, when comparing the genotypes within a treatment, AsA did not affect the nutrient uptake, and the differences in the nutrient levels were more closely related to the genotype than to the seed treatment. With respect to the toxicity of Al, the tolerant genotype DA did not show differences in Al uptake between treatments; nevertheless, the AsA seed treatment decreased Al uptake in Mo17 (from 215.30 to 173.80 mg of Al.Kg^-1^ dry matter).

**Table 3:** Nutrient and aluminum uptake in 50-day-old DA and Mo17 maize shoots.

		**Macronutrients (g.Kg** ^**-1**^ **)**					
**Genotype**	**Treatment**	**N**	**P**	**K**	**Ca**	**Mg**	**S**
**DA**	Unprimed	10.44 ± 0.40 aA	2.04 ± 0.20 aA	19.64 ± 1.02 aA	1.00 ± 0.06 aA	0.67 ± 0.03 aA	0.72 ± 0.01 aA
	Ascorbate	10.18 ± 0.30 aA	2.07 ± 0.10 aA	21.42 ± 1.59 aA	0.95 ± 0.23 aA	0.73 ± 0.07 aA	0.76 ± 0.03 aA
**Mo17**	Unprimed	10.91 ± 1.35 aA	3.02 ± 0.01 aB	16.45 ± 0.38 aA	0.75 ± 0.00 aA	0.50 ± 0.00 aB	0.97 ± 0.11 aB
	Ascorbate	11.10 ± 1.37 aA	2.81 ± 0.29 aB	17.21 ± 2.68 aA	0.78 ± 0.08 aA	0.55 ± 0.05 aB	1.02 ± 0.08 aB
		**Micronutrients (mg.kg** ^**-1**^ **)**				**Toxic element (mg.kg** ^**-1**^ **)**	
**Genotype**	**Treatment**	**Cu**	**Fe**	**Mn**	**Zn**	**Al**	
**DA**	Unprimed	3.33 ± 0.33 aA	70.67 ± 1.69 aA	69.83 ± 2.33 aA	22.00 ± 2.18 aA	99.33 ± 3.40 aA	
	Ascorbate	3.00 ± 0.29 aA	68.83 ± 5.95 aA	66.67 ± 2.24 aA	22.00 ± 2.02 aA	107.07 ± 4.88 aA	
**Mo17**	Unprimed	2.75 ± 0.75 aA	66.25 ± 1.75 aA	47.75 ± 3.75 aB	20.25 ± 0.25 aA	215.30 ± 8.80 bB	
	Ascorbate	2.25 ± 0.25 aA	60.75 ± 1.75 aA	50.50 ± 2.50 aB	23.25 ± 4.75 aA	173.80 ± 1.20 aB	

Different uppercase letters denote significant differences between the genotypes. The significant differences among seed treatments are shown using different lowercase letters (n = 3).

#### Lipid peroxidation and hydrogen peroxide

**Fig 7:**
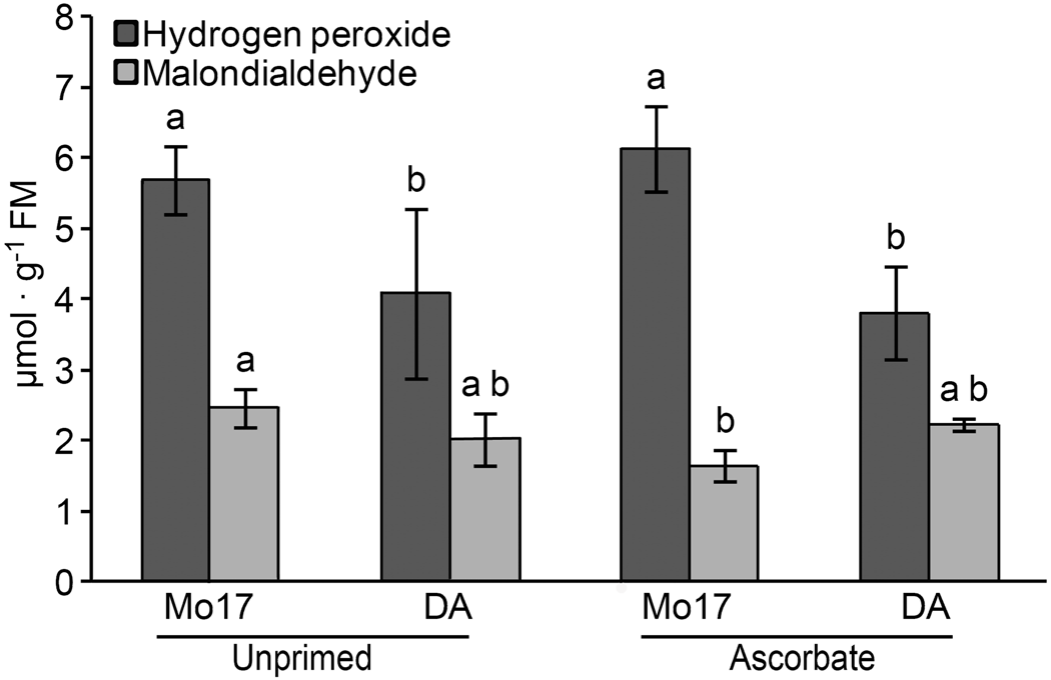
Hydrogen peroxide (dark gray) and malondialdehyde (light gray) quantification. The similar letters above the bars denote means that are not significantly different at a confidence level of 95%. FM = fresh matter.

Malondialdehyde (MDA), a product of lipid peroxidation, was measured as an indicator of oxidative damage to maize shoots (Fig 7). Seed priming reduced the production of MDA in Mo17 shoots (from 2.47 to 1.64 μmol MDA.g^-1^ fresh matter); however, this treatment did not change the lipid peroxidation rate in the tolerant DA genotype. The mean MDA production did not differ between the genotypes, but the production of hydrogen peroxide was significantly higher in Mo17 shoots compared with DA shoots (Fig 7).

#### Ascorbate determination

**Fig 8:**
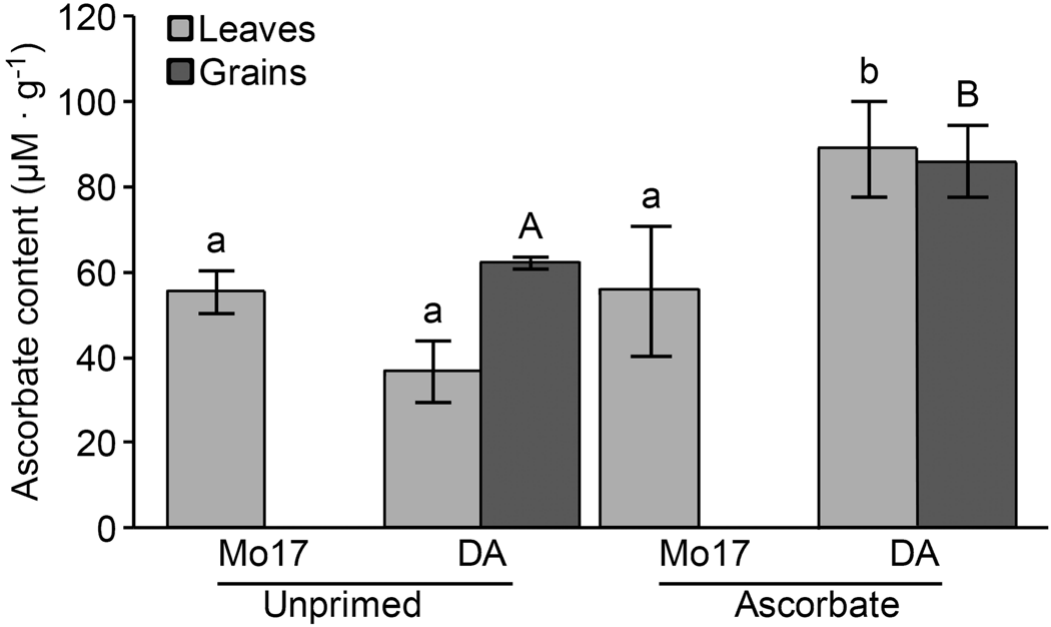
Spectrophotometric analysis of the ascorbate content of the maize leaves (Mo17; DA) and grains (DA). Similar letters above the bars denote means that are not significantly different at a confidence level of 95%. Ascorbate content in μM.g^-1^ fresh matter.

Seed priming increased ascorbate in DA from 36.9 to 89.10 μM ascorbate.g^-1^ fresh matter (Fig 8), whereas this treatment did not affect the ascorbate content of Mo17 leaves. The ascorbate content in the kernels was also quantified for DA, which exhibited a higher content in the plants that were treated with AsA (Fig 8).

#### Antioxidant enzyme activity

**Fig 9:**
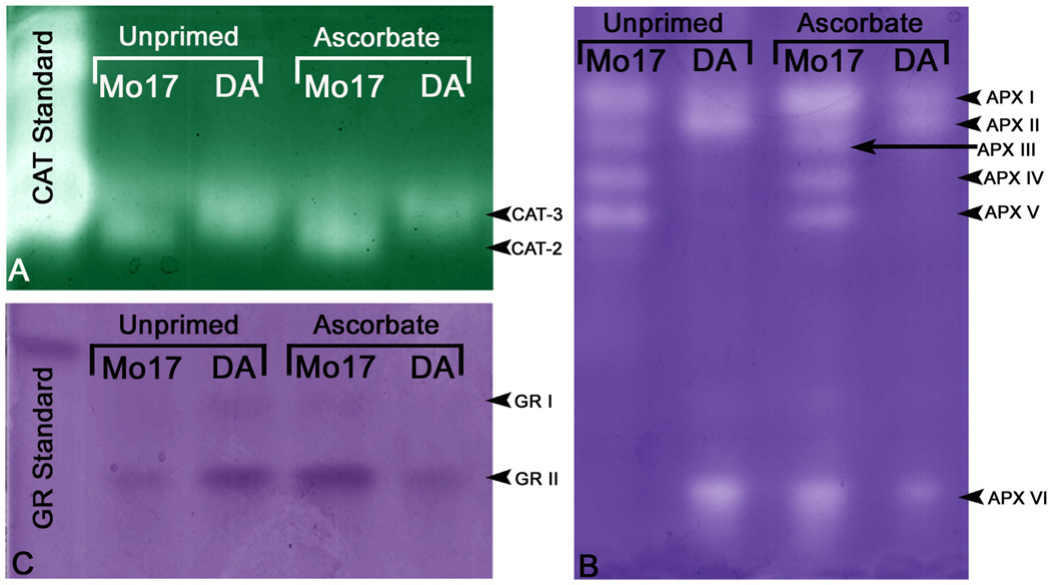
Visualization of enzyme activity in maize leaves determined from 10% polyacrylamide gels. (A) Visualization of catalase activity (CAT). (B) Visualization of ascorbate peroxidase activity (APX). (C) Visualization of glutathione reductase activity (GR).

The activity of the enzyme CAT changed with respect to seed priming. The Mo17 genotype exhibited two CAT isoenzymes that differed in activity levels, whereas the DA presented only one isoenzyme (Fig 9A). Based on the molecular masses that were observed by Scandalios [43], these isoenzymes were classified as CAT-2 (cytosolic or peroxisomal) and CAT-3 (mitochondrial). A densitometric analysis of the gels revealed that CAT-3 from the Al-tolerant genotype was relatively more active than CAT-3 from the Al-sensitive genotype (Table 3). Notably, seed priming significantly reduced CAT-3 activity in DA (from 55.03% to 41.87%; Table 3), while Mo17 exhibited an increased CAT-2 activity when primed (Fig 9A and Table 4).

**Table 4:** Densitometric analysis of catalase activity (CAT) in non-denaturing PAGE. Activities are presented as a percent (%) relative to a bovine standard.

Isoenzymes	Unprimed		Ascorbate	
	Mo17	DA	Mo17	DA
CAT-3	21.97 ± 0.74 a	55.03 ± 1.93 c	27.25 ± 1.78 a	41.87 ± 3.42 b
CAT-2	25.75 ± 2.01 a	0 -	38.59 ± 3.48 b	0 -

Similar letters denote means that are not significantly different at a confidence level of 95% (n = 2 gels).

Non-denaturing PAGE followed by APX activity staining revealed up to six distinct isoenzymes between the two genotypes (depending on treatment), which were marked as APX I, II, III, IV, V and VI (Fig 9B). Mo17 exhibited APX I, III, IV, V and VI, while DA exhibited APX I, II and VI. APX I was shown to increase activity in Mo17 treated with AsA, whereas APX VI was induced in Mo17 treated with AsA (Fig 9B). APX II and VI activities were reduced in the DA genotype with AsA treatment (Fig 9B; Table 5).

**Table 5:** Densitometric analysis of ascorbate peroxidase activity (APX) in non-denaturing PAGE. Activities are presented as a percent (%) relative to identified bands.

Isoenzymes	Unprimed		Ascorbate	
	Mo17	DA	Mo17	DA
APX I	22.16 ± 2.3 a	18.35 ± 0.43 a	23.27 ± 0.41 a	19.63 ± 1.14 a
APX II	--	23.13 ± 3.55 a	--	23.3 ± 0.29 a
APX III	16.35 ± 2.19 a	--	12.82 ± 2.08 a	--
APX IV	12.06 ± 3.85 a	--	8.77 ± 1.11 a	--
APX V	15.12 ± 4.5 a	--	7.93 ± 1.11 a	--
APX VI	5.84 ± 0.13 a	31.77 ± 0.9 b	24.6 ± 3.71 bc	18.15 ± 4.56 c

Similar letters in the same line denote means that are not significantly different at a confidence level of 95% (n = 2 gels).

With respect to the activity of GR, one clear GR isoenzyme (GR II) and possibly a second (GR I) with a much lower activity were observed (Fig 9C). Mo17 exhibited a major increase in GR II activity when subjected to AsA, whereas the exact opposite behavior was observed for the DA genotype (Fig 9C; Table 6).

**Table 6:** Densitometric analysis of glutathione reductase (GR) in non-denaturing PAGE. Activities are presented as a percent (%) relative to the standard.

Isoenzymes	Unprimed		Ascorbate	
	Mo17	DA	Mo17	DA
GR I	45.43 ± 0.12 a	45.58 ± 0.31 a	44.79 ± 0.86 a	45.8 ± 0.21 a
GR II	74.64 ± 8.53 a	80.99 ± 7.02 a	117.52 ± 14.48 b	74.15 ± 0.8 a

Similar letters in the same line denote means that are not significantly different at a confidence level of 95% (n = 2 gels).

**Fig 10:**
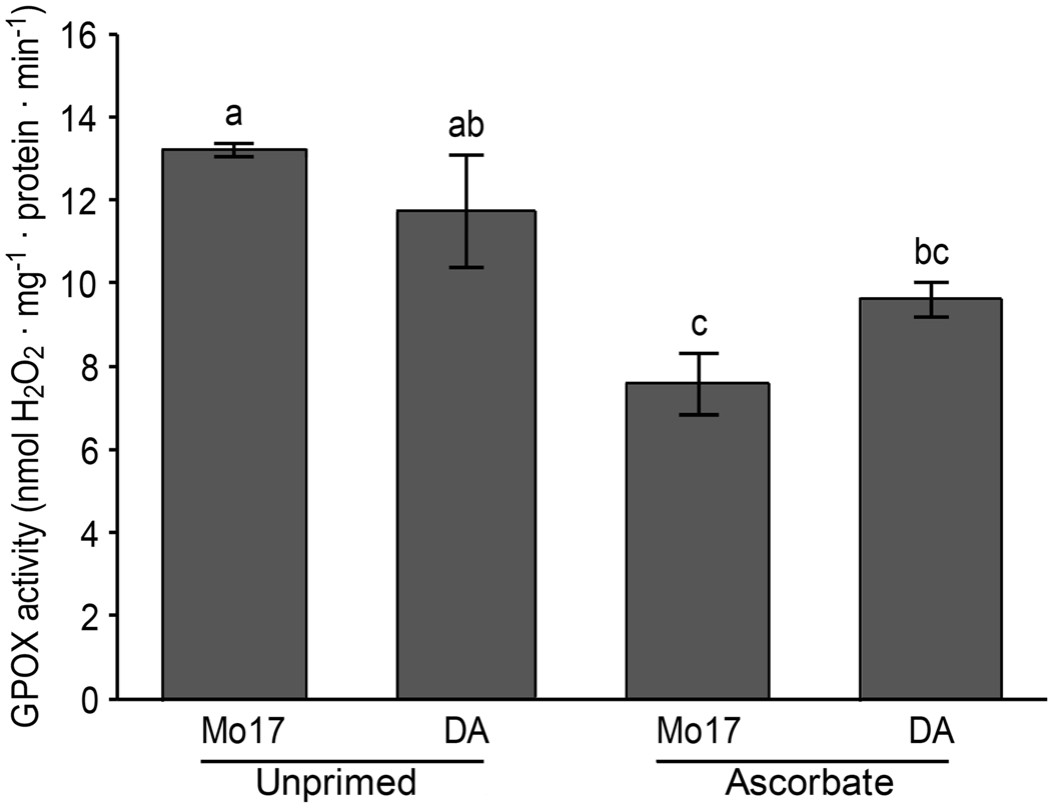
Spectrophotometric analysis of guaiacol-type peroxidase (GPOX) activity in maize leaves. Similar letters denote means that are not significantly different at a confidence level of 95%.

The total activity of GPOX was determined using a spectrophotometric assay. Ascorbic acid reduced GPOX activity (from 13.23 to 7.60 μmol H_2_O_2_.min^-1^.mg^-1^ protein) in Mo17, whereas no changes were observed in total GPOX activity in DA (Fig 10).

#### Klason lignin determination

The total lignin content was measured in 50-day-old maize shoots. The ascorbic acid treatment decreased the lignin content in Mo17 but not in the DA genotype. The data presented in Table 7 show that although levels of soluble lignin were not altered, the residual (or acid-insoluble) lignin content was reduced by AsA (from 10.01% to 8.81%). The reduction in total lignin in the Mo17 maize plants that originated from the primed seeds was 9.98% (Table 7).

**Table 7:** Lignin and holocellulose contents in shoots of 50-day-old DA and Mo17 maize plants (data in percent relative to 1 g completely dry material).

Genotype	Treatment	Soluble lignin	Residual lignin	Total lignin	Holocellulose
**DA**	Unprimed	2.93 ± 0.04 a	8.94 ± 0.09 a	11.87 ± 0.12 a	62.66 ± 0.61 a
	Ascorbate	2.86 ± 0.12 a	9.10 ± 0.23 a	11.96 ± 0.34 a	62.72 ± 0.43 a
**Mo17**	Unprimed	2.73 ± 0.08 a	10.01 ± 0.33 b	12.73 ± 0.41 b	62.40 ± 1.44 a
	Ascorbate	2.65 ± 0.05 a	8.81 ± 0.30 a	11.46 ± 0.35 a	62.10 ± 1.08 a

Observation: means with different letters within a column denote significant differences at a confidence level of 95% (n = 3).

## Discussion

Two main experiments were designed and carried out to verify the main effect of priming on three distinct maize genotypes. In the greenhouse experiment, AsA was shown to improve the root and shoot growth of the Al-sensitive B73 inbred maize line. A similar growth improvement was observed in the field for two other genotypes, Mo17 (Al-sensitive) and DA (Al-tolerant). When comparing B73 and Mo17, both characterized as Al-sensitive genotypes, the responses for growth enhancement and reduction in Al absorption to AsA-seed priming were similar. In the field experiment it was observed that the conversion of growth enhancement to yield improvement only occurred for the Al-tolerant genotype. AsA-priming improved early growth of Mo17 and DA plants (data obtained at 70 days after sowing), however Mo17 presented a general growth inhibition and died within 104 days after sowing, therefore grain productivity was only analyzed for DA. Seed priming with AsA increased the grain yield in the tolerant genotype. These new findings suggest that application of AsA enhances Al-tolerance. However, to provide Al-tolerance through the entire cycle of the sensitive Mo17, lower Al stress conditions should be tested in future experiments, and alternatively, higher amounts of AsA-priming might be studied.

In the greenhouse experiment, the AsA treatment improved the growth of maize roots and shoots (Figs 1 and 2). The shortest roots had the largest amount of absorbed aluminum (Fig 3). Therefore, AsA treatment reduced the uptake of Al by the roots of the Al-sensitive genotype B73, which can be explained by the increase in the expression of the key lignin gene *COMT*1 (Fig 4). The reduced Al uptake by the root cells could be related to the up-regulation of the lignin gene in the roots as previous studies have linked lignin to Al tolerance [23,50,63]. Ezaki et al. [23] proposed that lignin can form a complex with Al ions and therefore reduce the harmful effects of Al in the roots. Furthermore, lignin also has a role as a sink for ROS [23]. Under Al stress, the reduced expression of a possible repressor, *ZmMYB*31 [29], is in agreement with the increase in the *COMT*1 transcripts in the roots of AsA-primed seeds. Hence, it is suggested that this transcription factor is affected by seed priming and could be at least partially responsible for the increase in lignin gene expression in the roots. On the other hand, chelating Al in lignin would not result in a reduction of Al in the plant [23,64], therefore, it is implied that AsA is excluding Al in the sensitive line B73 because reduction of Al in roots was observed. In the work of Ezaki et al. [23], wild-type and transgenic *Arabidopsis thaliana* lines treated with 100 μM Al showed morin-specific fluorescent signals (Al staining) in their root tips. According to those authors, over-expression of another gene family involved in vesicle transport promotes efflux of Al.

In contrast, a different behavior of lignin gene expression was observed in the shoots. No differences were observed in Al absorption in B73 shoots. However, when the seeds were primed with AsA, the expression of *COMT*1 was reduced both with and without stress (Fig 5 and Table 1). When unprimed seeds were analyzed, the germination under stress caused the up-regulation of *COMT*1 expression. When the expression of the possible repressor *ZmMYB*42 was analyzed, it appeared that this transcription factor responded to Al stress in the shoots because there was a total reduction in expression that agreed with the induction of *COMT*1 under Al stress. The expression of *ZmMYB*42 was affected by AsA in both soil types, however, its repression under Al stress in shoots does not explain the repression of *COMT*1 by AsA, suggesting that further transcription factors are involved in repression of lignin gene in shoots.

The *COMT*1 expression in the shoots of AsA primed seeds that were germinated in low Al exhibited the lowest expression levels of the lignin genes among all treatments, as well as the highest expression levels of the transcription factors ZmMYB31 and ZmMYB42 when the two repressors were analyzed together, which agrees with the findings of Fornalé et al. [28], who presented evidence that these transcription factors down-regulate *COMT*1 in maize.

Therefore, in the greenhouse experiment, it can be suggested that the increase in lignin gene expression (*COMT*1) in the roots may underlie the reduction in Al uptake, a relationship that previous reports have elucidated [23,50,63]. However, in contrast, the reduction in *COMT*1 expression in the shoots may be due to the increased allocation of carbon to the roots for lignin biosynthesis (trade-off behavior).

A remarkable result was observed in the field experiment, specifically, seed priming with AsA resulted in increased grain yield by approximately 60% for the Al-tolerant genotype (DA), demonstrating the potential use of this treatment in improving crop performance (Table 2 and Fig 6). Therefore, in general, the results of the treatment with AsA were very positive for maize, demonstrating promising possibilities for future use.

With respect to plant growth, recent work demonstrated that seed priming with an AsA solution (2 mM) increased root and shoot lengths and the foliar area of wheat plants under drought conditions [37]. These authors suggested that this increase occurred due to a decrease in oxidative damage. In our study, the seed treatment using AsA powder also improved the growth of the Mo17 and DA plants that were germinated in soil with very high Al content (729 mmol_c_ Al.dm^-3^ soil). However, our study used powered AsA on dry seeds, differently than Farooq et al. [37]. Considering that approximately 1.8 mg was adhered to the surface of B73 seeds and one seed imbibed 0.1 g of water until constant weight, this is approximately equivalent to imbibing a seed in 100 mM. This concentration is much higher than those found in previous reports, assuming that 100% of adhered AsA entered the seed. Nevertheless, under the conditions tested in this study, the effect of AsA was not sufficient for the survival of the Al-sensitive line Mo17 under high Al-stress, showing that even though this treatment improved growth aspects, genetics dominated and played an apparent major role when yield is concerned.

The important observation of the significant reduction in Al uptake by Mo17 caused by the AsA treatment deserves further attention and research. In the field experiment, seed priming with AsA reduced Al accumulation in the shoots of Mo17 (Table 3), reducing the stress metabolite malondialdehyde (MDA) (Fig 7). This is supported by the observation for DA, in which Al uptake in the shoots and MDA production remained unchanged. These data are related to the activity of GPOX, a peroxidase involved in lignification [65–67], because the AsA treatment applied in this study decreased the GPOX activity in the shoots of Mo17 but not those of DA.

This study clearly indicates that different mechanisms are initiated by seed priming in Al-sensitive and Al-tolerant genotypes. In the case of Mo17, seed priming with AsA reduced Al uptake, whereas the Al uptake remained unchanged in DA. Therefore, the treatment appears to interfere with lignin synthesis in Mo17 (Table 6), whereas a different mechanism occurs in DA. The critical question raised from these data is that if either lignin synthesis or Al uptake are not altered in DA, how does this treatment improve the growth of this tolerant genotype?

One hypothesis is that the powdered AsA that was used may have adhered to the surface of the seeds and was consequently diluted in the soil water, generating ascorbate anions and hydrogen protons in the soil water. Therefore, infiltration of AsA into the seed may have occurred when it was planted in the soil and may have also changed the interactions between soil microorganisms and the maize plants. Because the soil water diluted the AsA present on the seed, the magnitude of the influence of AsA on microorganisms and the amount that entered the seed should be investigated in future studies. Moreover, some studies have demonstrated certain beneficial effects of microbes that induce tolerance to abiotic stresses [68,69]. There is also evidence that microorganisms ameliorate the scavenging capacity of ascorbate in beans [70]; however, more studies are needed to confirm this hypothesis and identify which microorganisms are involved in maize root interactions and determine how these microorganisms are affected by AsA.

Based on the increase in the ascorbate content in the DA leaves (Fig 8), we may also suggest that because ascorbate is a cofactor for a number of important enzymes, such as proline and lysine hydroxylases, which are involved in the synthesis of extensins (important glycoproteins for cell wall expansion) [71], the increased ascorbate content in DA leaves may be directly involved in the growth improvement of this genotype.

In attempting to understand the effect of AsA on the Al-tolerant genotype, it is important to bear in mind that plants produce AsA in the mitochondria via L-galactose [72], D-galacturonic acid [73] and D-glucuronic acid, which are generated from *myo*-inositol [74]. Based on the work conducted by Horemans et al. [75], AsA is present in several cell compartments, for example, the chloroplasts, cytosol, vacuoles and apoplast. Because AsA biosynthesis does not occur in the apoplast, the presence of AsA transporters has been suggested to play an important role in the translocation of AsA from the cytosol to the apoplast. Moreover, subsequent studies have shown that ascorbate is also transported in the reverse direction (from the apoplast to the cytoplasm) ([75] and references therein). The transport of AsA from the apoplast to the cytoplasm could explain why the activity of CAT-3 and APX VI were reduced by the treatment used, even though we did not observe a reduction in H_2_O_2_ in the DA genotype.

There are three catalase isoenzymes (CAT-l, CAT-2 and CAT-3) in maize that are encoded by three distinct and independent genetic loci (*Cat*1, *Cat*2 and *Cat*3) [76]. Their activity is tissue-specific, for example, CAT-1 is not found in mature maize leaves, although this isoenzyme is present in the pericarp and endosperm. CAT-3 is most active in the maize stem, whereas in leaves, two isoenzymes (CAT-2 and CAT-3) exist, although only one, CAT-3, is present in some genotypes [76]. In this study, two isoenzymes were observed in the leaves (CAT-2 and CAT-3) of Mo17, while only CAT-3 was found in DA, which was more active than in Mo17 (Table 3 and Fig 9A). This suggests that CAT-3 may play an important role in Al-tolerance in the DA genotype because in this genotype, the H_2_O_2_ content was lower than in Mo17, a possible consequence of the higher CAT-3 activity in DA than in Mo17.

In addition to the action of CAT, H_2_O_2_ may also be converted to water via “peroxidases”, a term denoting the enzyme-catalyzed reduction of H_2_O_2_ and the oxidation of a variety of substrates [44]. The class I peroxidase family includes the intracellular enzymes originating from prokaryotes, cytochrome *c* peroxidase, catalase-peroxidase and ascorbate peroxidase (APX) [44]. The latter, the focus of this study, oxidizes ascorbate to form dehydroascorbate (DHA) and water [77].

Non-denaturing PAGE analysis revealed six potentially true APX isoenzymes, which could be located in different compartments of the cell (Fig 9B). In this study, they were denominated by APX I, II, III, IV, V and VI, according to their electrophoretic mobility and Mo17 contained more isoenzymes than DA. Mo17 contained APX I, III, IV, V and VI while DA contained APX I, II and VI. Based on the gel analysis, DA had one isoenzyme that was not present in Mo17, i.e., APX II, which could also be involved in tolerance to Al shown by the DA genotype.

Another observation obtained from these data is that APX VI appeared to respond to AsA treatment more than the others isoenzymes, but different responses were observed in the different genotypes. APX VI was induced by AsA in Mo17, while in the DA genotype, APX VI activity was reduced with AsA (Table 4). When comparing the responses of the GR II and APX VI activities, the striking similarity is extremely relevant (Table 5 and Fig 9C). For instance, the Al-tolerant genotype DA exhibited a reduction in GR II and APX VI activities caused by the AsA treatment. The activity of APX consumes AsA and generates dehydroascorbate (DHA), which can be regenerated into AsA via an enzymatic reaction mediated by dehydroascorbate reductase (DHAR) using glutathione (GSH) as a reducing substance [78]. Glutathione reductase (GR) plays an important role in the antioxidant defense machinery by reducing the oxidized form of glutathione (GSSG), thereby regenerating GSH [79]. It is possible that at low levels, AsA is involved in the enzymatic antioxidant machinery (with APX) and at certain levels, it can regulate transcription factors and thereby reduce enzymatic activity. In the DA genotype, the reduction in GR II activity can be explained by the reduction in APX VI activity in seeds treated with AsA.

The AsA content increased in DA leaves as a result of the seed treatment. This new finding suggests that in Al-tolerant maize, AsA may signal the down-regulation of genes encoding CAT-3 and APX VI to reduce their activities, possibly because AsA already acts as a non-enzymatic antioxidant and it is energetically unnecessary for the CAT-3 and APX VI enzymes to function in this context. The role of AsA in the regulation of transcriptional factors has previously been reported in humans with respect to the ascorbate stimulation of collagen gene expression [80] as well as the down-regulation of a transcription factor involved in tumor growth and apoptosis [81]. Additional studies are needed in plants, although AsA appears to be associated with flowering time, developmental senescence, programmed cell death, and responses to pathogens [82,83].

The foliar AsA content did not change in Mo17. However, the GPOX activity was reduced in this genotype but did not change in DA (Fig 10). GPOX plays a critical role in plant land colonization because in addition to adapting the organisms to a more oxygenated environment, this enzyme also provides more rigid structures [66], which is important for lignin in the context of this study. Hydrogen peroxide is used to oxidize monoligonols during the process of lignin polymerization [65]. The lignin polymers grow following the coupling of the monolignols in the “nucleation sites”, which are dependent on peroxidase reactions occurring in the middle lamella and the corners of the cell [84]. In maize, GPOX may be located at the apoplastic surface of the plasma membrane of root maize cells as demonstrated previously [85]. The reduction in GPOX activity in Mo17 shoots agrees with the reduction in total lignin content observed for this genotype.

Regarding nutrient uptake, a comparison between Mo17 and DA indicated that the tolerant genotype absorbed less phosphorus (P) and sulfur (S) than did Mo17. Sulfur and P are essential elements for plant growth. Thus, this result was unexpected because DA showed better growth than Mo17, most likely because the use of these nutrients in tolerant plants is more efficient than in sensitive plants. Genotypic differences in S use efficiency during the vegetative stage have been reported for mustard [86] and canola [87]. Moreover, substantial differences in P utilization have been shown in maize [88,89]. Furthermore, some studies have linked P use efficiency to Al tolerance [1]. Therefore, in addition to Al tolerance, DA appears to be S and P efficient, characteristics that are highly desirable, especially with respect to P, given the limitation of mineral phosphate fertilizers [89].

The differences in nutrient uptake were also shown to be more correlated with genotype than with the AsA treatment. When comparing the genotypes within a treatment, DA absorbed more magnesium (Mg) and manganese (Mn) than did Mo17; Mg and Mn are essential nutrients for photosynthesis. Mg^2+^ is the central atom of the chlorophyll molecule, and fluctuations in its levels in the chloroplast regulate the activity of key photosynthetic enzymes [90], whereas Mn^2+^ is involved in photosynthetic water oxidation to produce 1 molecule of oxygen, 4 electrons and 4 protons [91].

In summary, the AsA-seed treatment reduced the activities of the oxidative stress-related enzymes CAT-3 (mitochondrial catalase), APX VI (unknown compartment) and GR II (cytoplasm) in the Al-tolerant maize genotype, while in the Al-sensitive genotype, GPOX activity decreased, CAT-2 activity increased, APX VI and GR II were induced.

Therefore, AsA seed treatment presented some interesting effects that could be economically explored, such as the yield improvement in the Al-tolerant genotype. However, the AsA seed treatment did not induce Al tolerance in the entire life cycle of Al-sensitive genotype and did not improve nutrient absorption, although Al absorption was reduced. More studies are needed to verify whether this response is dose-dependent by using different amounts of AsA. Additional tests involving additional environments and other tolerant maize genotypes are also required and will most likely contribute to a better understanding of the interactions tested in this study.

## Supporting Information

S1 Fig Length of roots and shoots of 5-day-old maize seedlings (B73). (TIF)

S2 Fig *COMT*1, *ZmMYB*31 and *ZmMYB*42 expressions in the roots and shoots of 5-day-old maize seedlings (B73). (TIF)

S3 Fig Quantification of aluminum in roots and shoots of 5-day-old maize seedlings (B73). (TIF)

S4 Fig Nutrient and aluminum uptake in 50-day-old DA and Mo17 maize shoots. (TIF)

S5 Fig Productivity parameters for the maize genotypes. (TIF)

S6 Fig Densitometric analysis of catalase activity (CAT). (TIF)

S7 Fig Densitometric analysis of glutathione reductase (GR). (TIF)

S8 Fig Densitometric analysis of ascorbate peroxidase activity (APX). (TIF)

S9 Fig Spectrophotometric analysis of guaiacol-type peroxidase activity (GPOX). (TIF)

S10 Fig Lignin and holocellulose contents in shoots of 50-day-old DA and Mo17 maize plants. (TIF)

S11 Fig Ascorbate quantification. (TIF)

S12 Fig Hydrogen peroxide quantification. (TIF)

S13 Fig Malondialdehyde quantification. (TIF)
