# Supplementary figures and images for: Dry Priming of Maize Seeds Reduces Aluminum Stress
Source: PLoS One. 2015 Dec 29;10(12):e0145742. doi: 10.1371/journal.pone.0145742 (PMC4694655; doi:10.1371/journal.pone.0145742)

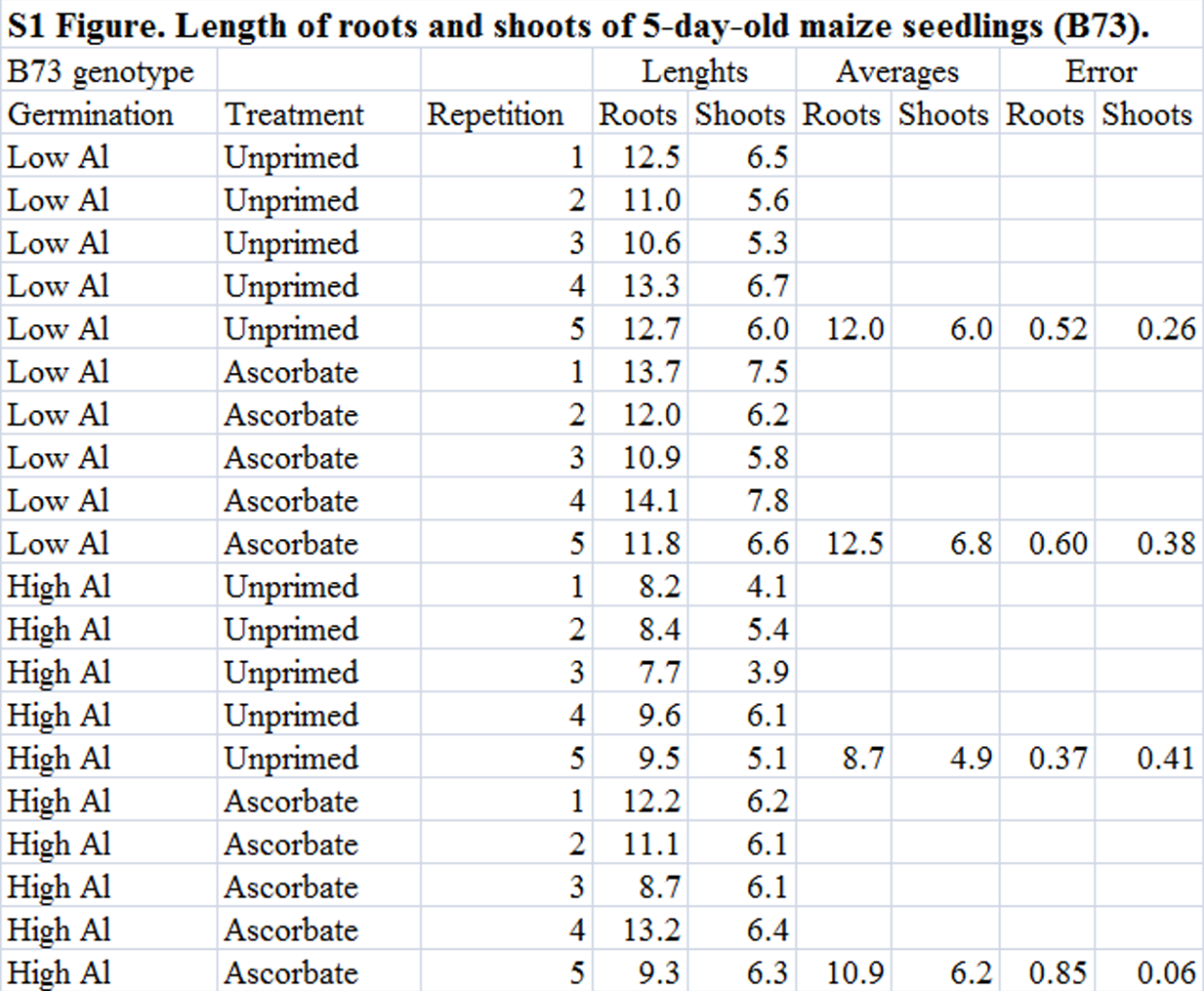

Supplement: S1 Fig — (TIF) [file pone.0145742.s001.tif]

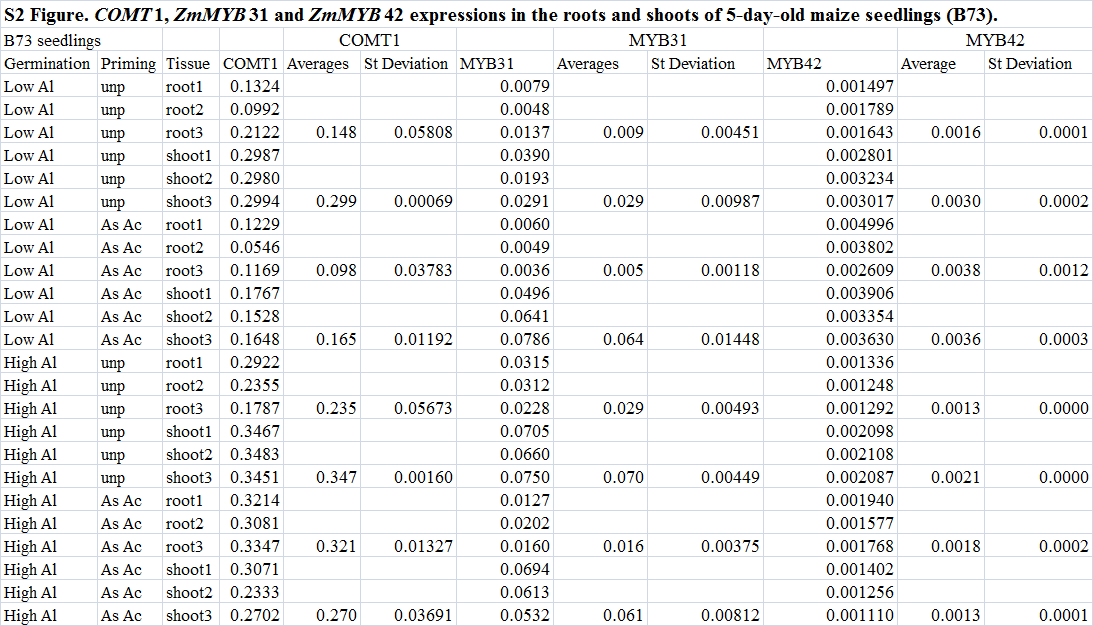

Supplement: S2 Fig — (TIF) [file pone.0145742.s002.tif]

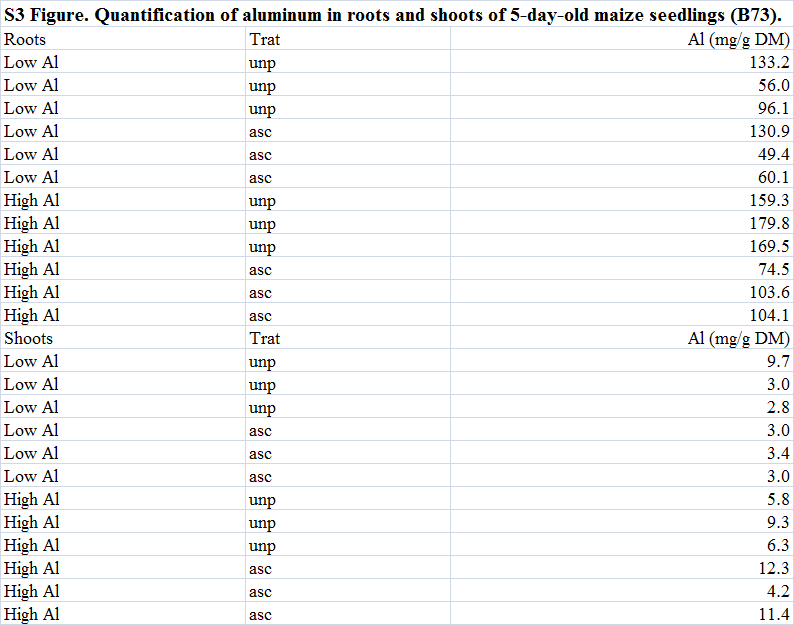

Supplement: S3 Fig — (TIF) [file pone.0145742.s003.tif]

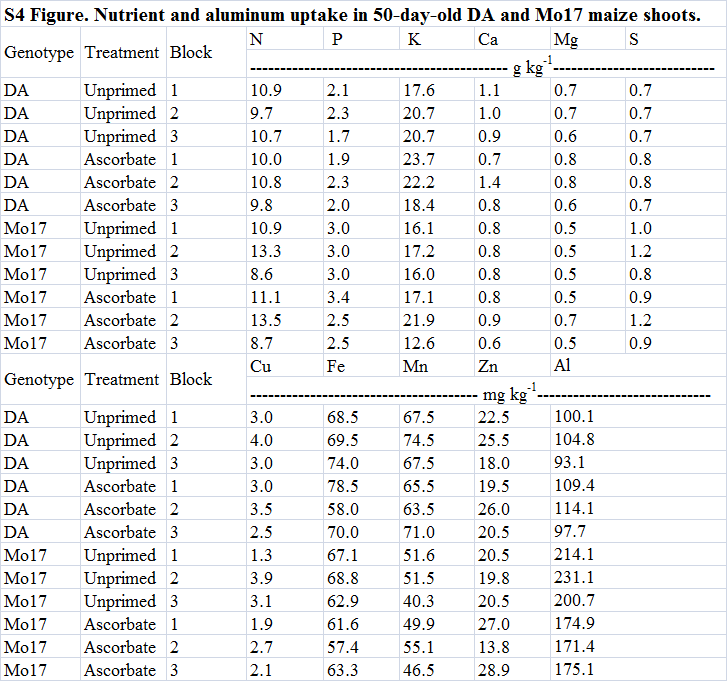

Supplement: S4 Fig — (TIF) [file pone.0145742.s004.tif]

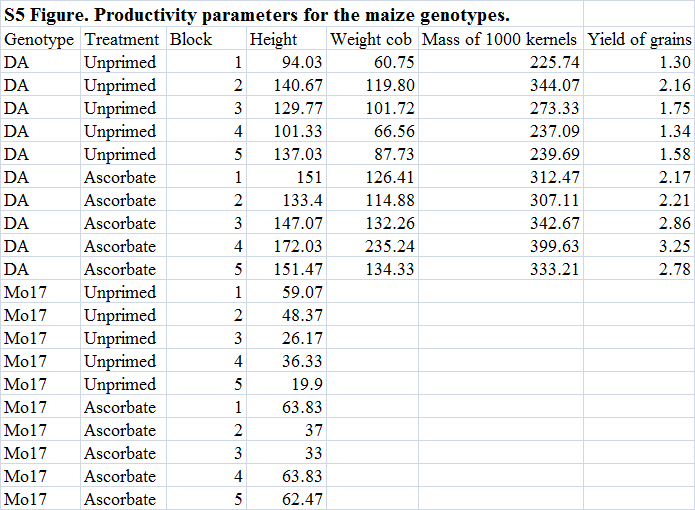

Supplement: S5 Fig — (TIF) [file pone.0145742.s005.tif]

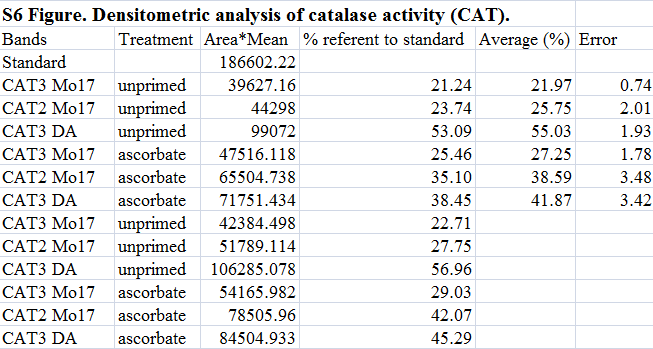

Supplement: S6 Fig — (TIF) [file pone.0145742.s006.tif]

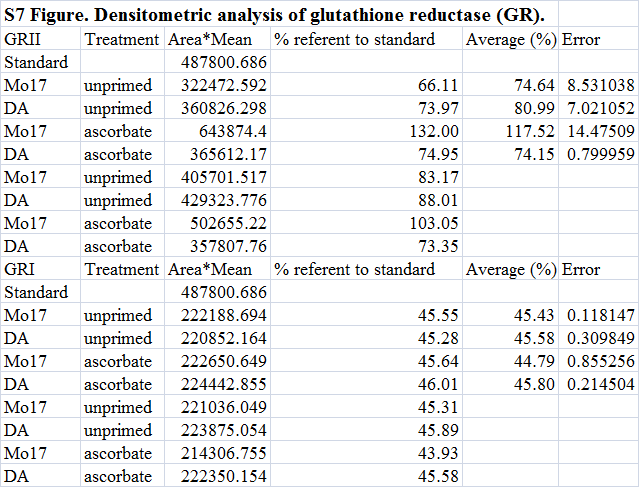

Supplement: S7 Fig — (TIF) [file pone.0145742.s007.tif]

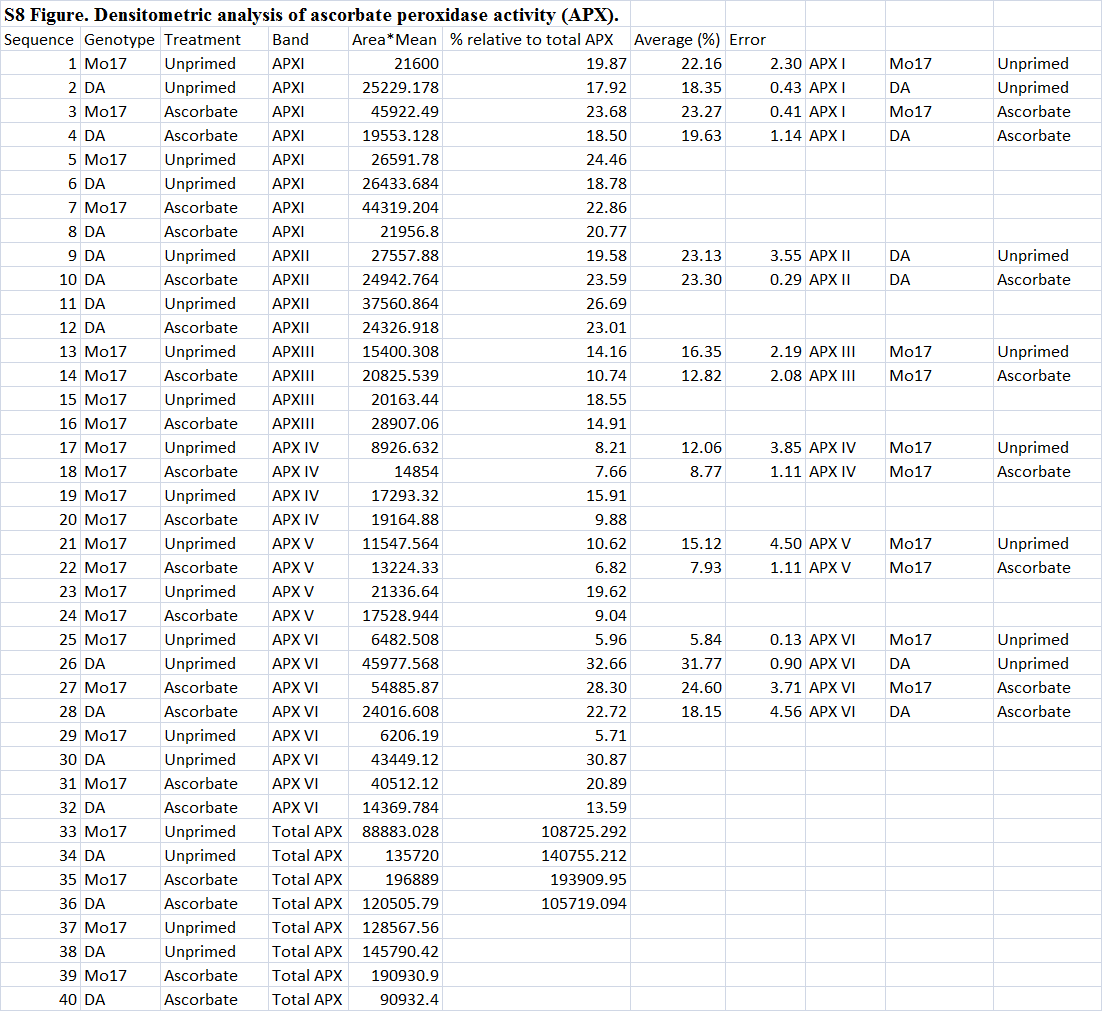

Supplement: S8 Fig — (TIF) [file pone.0145742.s008.tif]

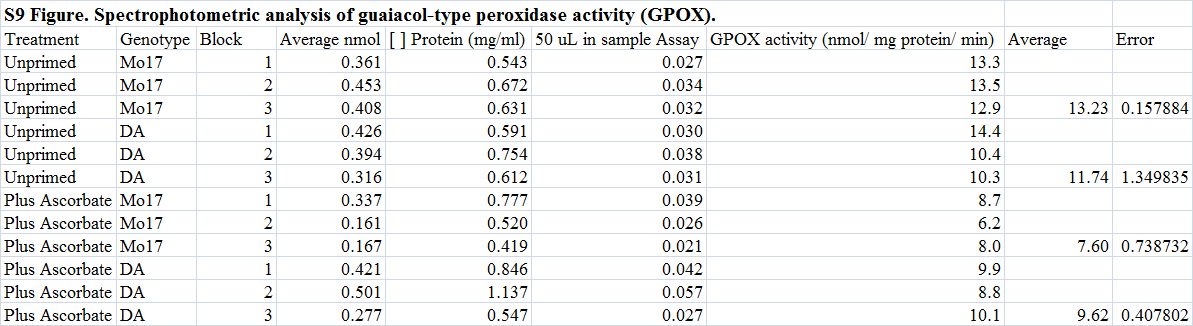

Supplement: S9 Fig — (TIF) [file pone.0145742.s009.tif]

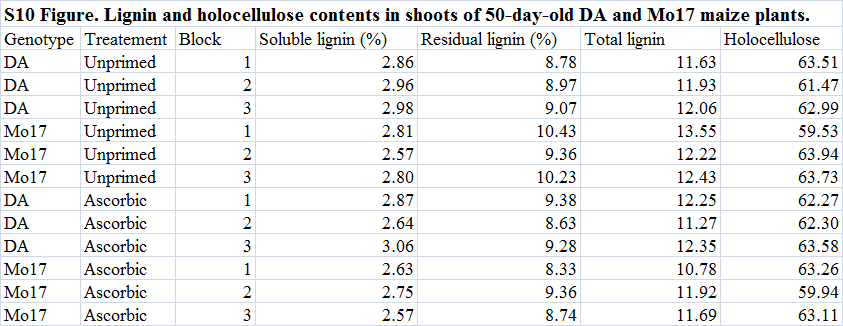

Supplement: S10 Fig — (TIF) [file pone.0145742.s010.tif]

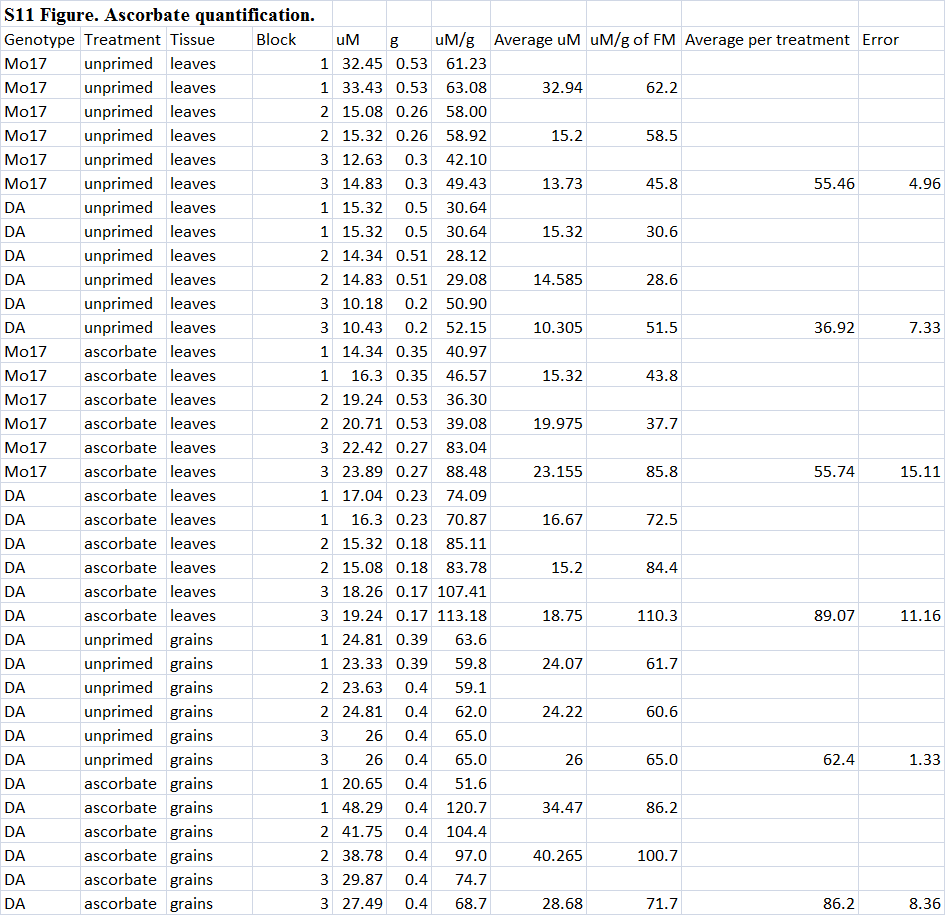

Supplement: S11 Fig — (TIF) [file pone.0145742.s011.tif]

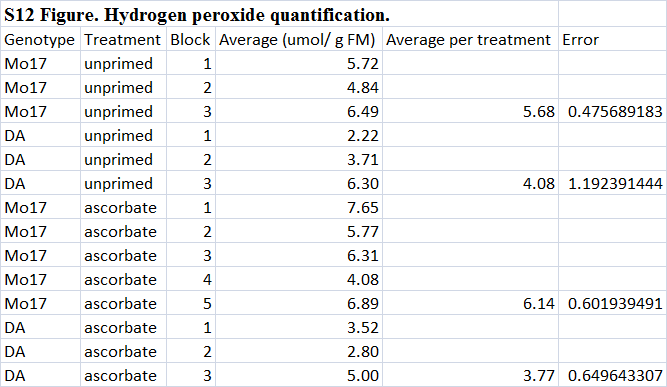

Supplement: S12 Fig — (TIF) [file pone.0145742.s012.tif]

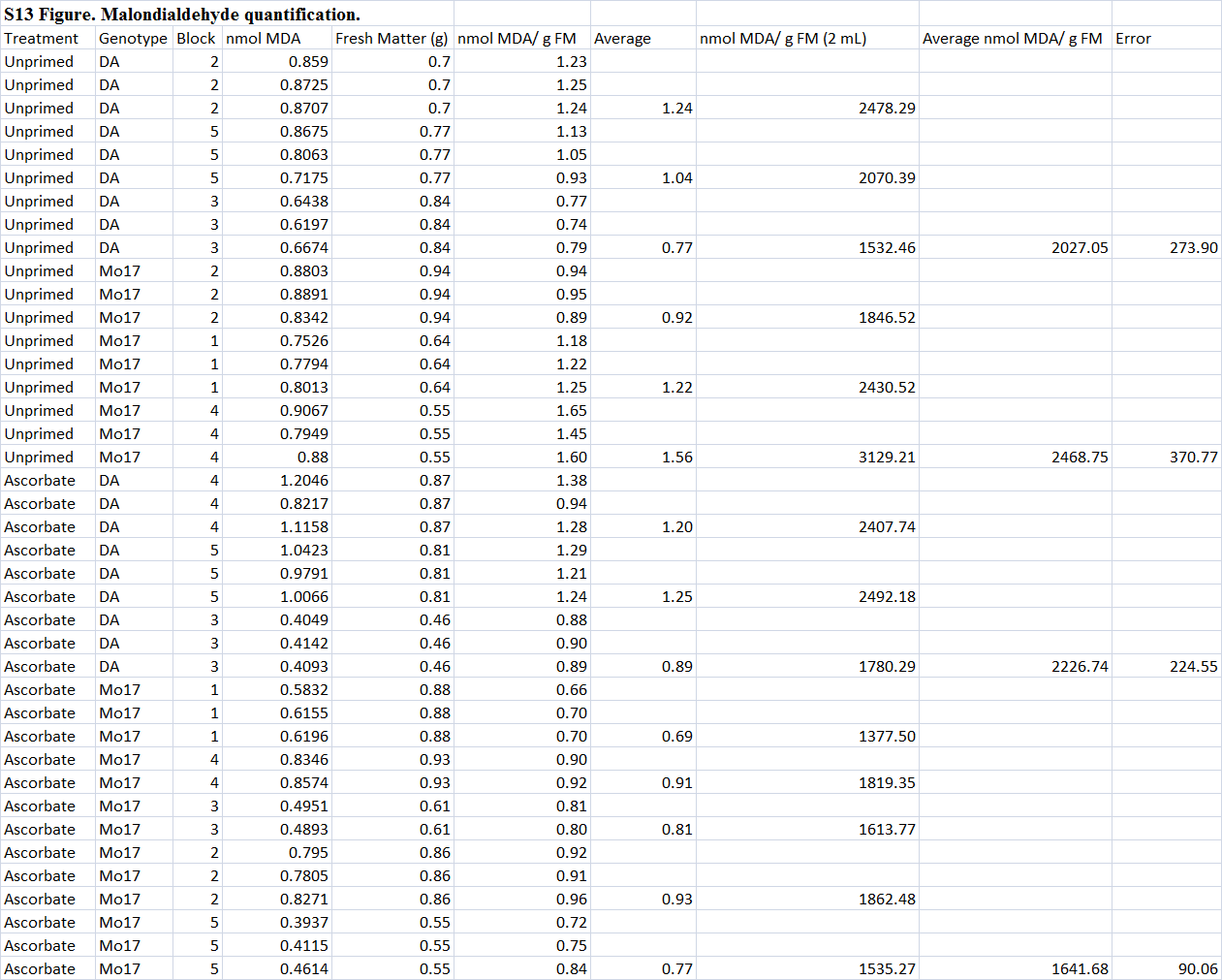

Supplement: S13 Fig — (TIF) [file pone.0145742.s013.tif]
